# The autonomic-ventricular coherence index: a novel single-lead ECG method with acquisition-dependent discrimination

**DOI:** 10.3389/fdgth.2026.1964571

**Published:** 2026-10-14

**Authors:** Jayasree R, Chemmalar Selvi G

**Affiliations:** School of Computer Science Engineering and Information Systems, Vellore Institute of Technology Deemed to be University, Vellore, India

**Keywords:** autonomic nervous system, cardiac coherence method, ECG, electrocardiogram, heart rate variability, biomarkers

## Abstract

**Background:**

Autonomic regulation and the heart's electrical activity are both central to normal cardiac function, yet there is currently no direct way to capture the interaction between them from a standard ECG. This paper introduces the Autonomic–Ventricular Coherence Index (AVCI), a single-lead ECG method that measures the beat-to-beat spectral coherence between variability in RR intervals and variability in QRS area.

**Methods:**

AVCI was developed and evaluated on 74 recordings from three PhysioNet databases (MIT-BIH Normal Sinus Rhythm, *n* = 18; BIDMC Congestive Heart Failure, *n* = 15; and MIT-BIH Arrhythmia, *n* = 41), with a further six recordings from the MIT-BIH Long-Term ECG Database used for preliminary independent validation. Coherence was integrated over the high-frequency band (0.15–0.40 Hz). Because the source databases differ in sampling rate and recording length, AVCI was examined both under each database's native recording conditions and after acquisition standardization, harmonizing all recordings to a common 128 Hz and 30-minute duration, additionally upsampling to 360 Hz for 30 min under uniform acquisition, and applying a two-tier duration-stratified framework. The analysis code is available at https://github.com/JayaSreejai/AVCI.git

**Results:**

Under native conditions, AVCI demonstrated a high capacity to distinguish between congestive heart failure and normal sinus rhythm (AUC 0.844). Like SDNN, it performed moderately in harmonized acquisition (NSR vs. CHF AUC 0.647; NSR vs. all-disease AUC 0.619). Under native conditions, machine learning models trained on balanced datasets performed well (KNN accuracy 79.5%, ROC-AUC 85.7, SVM accuracy 79.0%, ROC-AUC 83.0), whereas under harmonized conditions, they produced average results. R-peak identification was consistent across QRS windows and accurate (F1 0.892). These results show that AVCI's diagnostic performance depends on acquisition parameters, particularly sampling rate and recording duration.

**Conclusions:**

AVCI is a practical and understandable measure of autonomic-ventricular coupling from a single ECG lead. Combined with machine learning, it offers a promising path toward more robust and explainable cardiovascular diagnostics. Standardized acquisition protocols are key to reliable clinical use.

## 1 Introduction

Cardiovascular Disease (CVD) is a disease that affects the normal functioning of the heart (cardiac) and blood vessels (vascular). Early, common, and often unnoticed symptoms include chest discomfort, palpitations, dizziness, and swelling in the hands and feet. The latter, severe symptoms require immediate medical attention, such as intense chest pain, numbness, sudden loss of consciousness, vomiting, and shortness of breath at rest. According to the World Health Organization (WHO), the leading cause of death at the global level is CVD, with nearly 17.9 million deaths (1). The India State-Level Disease Burden Initiative, led by the Indian Council of Medical Research (ICMR), reported that cardiovascular diseases contributed 28.1% of total deaths in India in 2016, up from 15.2% in 1990. Furthermore, ischemic heart disease was identified as the leading individual cause of disease burden in the country (2).

Diagnostic methods for CVD are usually performed through a grouping of clinical assessment, imaging, and laboratory investigations. These diagnostic methods are classified into three categories: non-invasive, invasive, and minimally invasive. The invasive methods are carried out with the help of an instrument inserted into the body (blood vessels), such as coronary angiography. Without inserting any instrument into the body, electrocardiography (ECG), blood pressure monitoring, clinical assessment, cardiac CT, cardiac MRI, sensors, and wearable devices such as smartwatches are non-invasive methods. The troponin (3) Cholesterol tests are both invasive and non-invasive tests. The patient's recovery after the invasive procedure takes a long time. Pain, discomfort, and swelling of the blood vessels can occur during needle insertion for blood tests. Irritation and allergies are caused by the gel applied during an ECG. These are some common diagnostic challenges. A traditional diagnostic tool focuses on recording the heart's physical structure, rhythm, and mechanical function. Some of them are the electrocardiogram (ECG), Echocardiogram (echo), stress test, and cardiac CT and MRI. Of these approaches, biomarkers are important because they provide quantifiable measures of physiological or pathological processes.

Biomarkers (4) are measurable biological indicators in the body. They are widely used for diagnosing diseases, assessing disease risk, and monitoring the body's response to treatment. These are biological “red flags” found in the blood that indicate heart stress or damage. There are numerous types of biomarkers, such as diagnostic, prognostic, monitoring, pharmacodynamic, and genetic. These include cardiac troponin, B- type natriuretic Peptide, C-reactive protein, and Lipid panel. Traditional biomarkers are generally derived from laboratory-based biological measurements such as blood proteins and biochemical indicators. The potential of digital biomarkers to enable ongoing, non-invasive health monitoring has attracted considerable attention. Digital biomarkers are quantifiable measurements obtained from digitally recorded physiological or behavioral data. Heart Rate Variability (HRV) (5–8) is a widely used non-invasive digital biomarker for monitoring and prognosis. It measures the relationship between heartbeats. It reflects autonomic nervous system activity. Standard measures of the system, such as SDNN, RMSSD, and LF/HF spectral power, are well-established markers of sympathovagal balance. However, HRV considers only the temporal variability of inter-beat intervals and fails to capture the coupled interaction between autonomic input and beat-to-beat changes in ventricular morphology. Circumstances such as congestive heart failure (CHF) and ventricular ectopy are known to interrupt this autonomic-ventricular coupling through mechanisms including autonomic remodeling, increased sympathetic tone, and altered ventricular activation patterns (7, 9, 10). To address this limitation, recent research has focused on advanced analytical techniques such as coherence analysis. Coherence measures how strongly two signals are related (synchronized) at different frequencies. These methods analyze the “synchronization” and “harmony” between body systems. Coherence is used to distinguish the various parts of cardiac activity. A regular pattern of signals characterizes normal cardiac function, whereas diseased conditions are considered to have disturbed or abnormal coherence, which can lead to myocardial infarction (11). Hence, coherence-based features are sensitive biomarkers that can be used to identify subtle abnormalities that might not be revealed in conventional analysis.

The degree of linear coupling at each frequency, normalized from zero (no coupling) to unity (perfect coupling), is quantified by the spectral coherence between two physiological time series. In cardiovascular signal analysis, coherence-based indices have demonstrated diagnostic utility, including wavelet phase coherence of cardiovascular oscillations (12, 13). Intra-QT coherence as a marker of ventricular tachycardia (14) and cross-spectral identification of myocardial infarction (11). Coherence within the high-frequency (HF) autonomic band indicates the degree to which respiratory-rate autonomic modulation and beat-to-beat ventricular variation covary when applied to RR-interval variability and QRS-morphology variability. This relationship is anticipated to differ significantly between healthy and diseased hearts.

In recent years, the field of artificial intelligence (AI) has taken another step forward in cardiovascular disease (CVD) diagnosis. For cardiovascular risk prediction and patient classification, structured clinical data have been successfully applied to classical machine-learning (ML) algorithms like logistic regression, decision trees, random forests, and support vector machines (15, 16). Deep learning (DL) methods are effective for arrhythmia and CHF detection and for real-time monitoring with wearable devices (9, 17, 18). However, while traditional machine learning approaches require handcrafted features, deep learning models may have less clinical interpretability. Moreover, these methods do not explicitly include the physiological coherence and connectivity of cardiovascular function (15, 19). Such frameworks can be enhanced by incorporating physiologically meaningful coherence-derived features to enable more clinically relevant and physiologically meaningful analysis of the cardiovascular system. This integration will make the model more explainable and robust, enhancing the accuracy of cardiovascular abnormality diagnosis with greater certainty. Coherence-guided AI systems are an important improvement in creating intelligent and explainable cardiovascular healthcare systems, providing more clinically applicable predictions. The main objective of this research is to overcome the limitations of traditional systems by developing a coherence-based framework to improve cardiovascular disease analysis. It meets the need for smart health care solutions in the modern world. The proposed approach combines coherence-based physiological parameters and AI-built computational models to improve accuracy, reliability, and diagnosis, thereby benefiting clinical decision-making.

In this study, we propose the Autonomic–Ventricular Coherence Index (AVCI), a novel single-lead ECG method based on spectral coherence. Autonomic regulation and ventricular electrical activity are physiologically fundamental but are not usually quantified together as a coupled signal in clinical ECG. AVCI quantifies this relationship as a computational coherence measure between RR-interval variability and QRS-area (morphology) variability. The proposed method is assessed using statistical validation and machine-learning classifiers, and because the source databases differ in acquisition, it is evaluated under both native and acquisition-standardized conditions. As a proof of concept, the framework may inform the future development of accessible single-lead cardiac screening tools. The primary contributions of this study are:

- The introduction of AVCI, a novel single-lead ECG method that jointly represents autonomic (RR-interval) and ventricular (QRS-morphology) dynamics through their frequency-domain coherence, providing an interpretable computational measure derived from a single lead. The interpretation of this coherence as autonomic–ventricular coupling is presented as a physiological hypothesis.
- A feature-extraction method with no explicit separation of Q, S, and T wave components and without loss of coherence information through a beat-masking approach. This further tests the behavior of the measure when acquired across different sampling rates and recording durations using standardized acquisition.
- A well-designed evaluation demonstrating that AVCI discriminates cardiac pathologies from normal sinus rhythm in a native recording environment, as well as the transparent assessment of the extent of the discrimination in acquisition-standardized environments.
- Evaluation in three clinical categories, native versus harmonized, within-database evaluations, and assessment of effect size and diagnostic performance using benchmark PhysioNet databases.
- Comparisons to traditional heart rate variability (HRV) measurements, demonstrating that AVCI does not appear to retain its apparent edge with native conditions, but after acquisition standardization with traditional HRV. Characterization of the conditions under which the two differ.
- Validating the AVCI feature on several supervised machine-learning classifiers, with reporting of balanced performance metrics that are suitable for class imbalance, both under native and standardized conditions.

The succeeding sections of this paper are structured as follows. Section II examines the relevant literature papers. Section III describes the methodology. Section IV presents the results. Section V presents the discussion, and Section VI completes the paper and highlights future research directions.

## 2 Related works

The development of single-lead ECG-based biomarkers for cardiovascular health assessment has been influenced by four major research domains: (i) heart rate variability (HRV) as an indicator of autonomic regulation, (ii) coherence-based physiological metrics, (iii) machine learning and deep learning techniques for ECG analysis, and (iv) ventricular repolarization and morphology-based biomarkers. This section reviews demonstrative studies in these areas and identifies the limitations of existing approaches that motivate the development of the proposed AVCI.

### 2.1 Autonomic biomarker (HRV)

HRV has received considerable attention as a digital biomarker in recent years due to its potential applications in health monitoring and clinical practice. However, there is no universal standard for HRV data collection and analysis, which can affect consistency across studies (5). There is considerable evidence for the clinical usefulness of HRV. In general, patients with CAD have reduced HRV, indicating autonomic impairment, as reported by (7). Likewise, HRV parameters that correlate with heart rate are more predictive of sudden cardiac death than traditional HRV parameters, suggesting that HRV is an important tool for assessing cardiovascular risk and overall heart health (6). Although benefits exist, HRV primarily measures changes in heartbeat timing and offers little insight into the effects of autonomic control on ventricular electrical activity. As a result, appropriate information about the relationship between the autonomic nervous system and the heart's electrical activity may be ignored. This limitation underscores the need to develop new biomarkers that reflect both autonomic and ventricular electrical function for a more complete assessment of cardiac health.

### 2.2 Coherence-based methods

Spectral coherence offers insights into physiological connections that single-signal HRV cannot detect. Sinha and Das use cross-spectral coherence in 12-lead ECGs to detect and focus on a myocardial infarction by using a support vector machine classifier. ECG cross-spectral coherence contains important pathological information (11). Intra-QT spectral coherence was introduced, calculated between the Q-wave-to-T-peak and T-peak-to-T-end segments, as a marker for the risk of sustained ventricular tachycardia (14). Wavelet-phase coherence between heart rate variability and blood flow oscillations to identify mechanisms across different types of exercise (12). Together, these studies show that coherence is a valuable tool for cardiovascular analysis. However, each method either links two segments of the same waveform, which requires reliable T-wave detection (14). Connects the ECG with another physiological signal (12) or uses multi-lead recordings (11). So far, no one has examined the coherence between RR-interval variability and QRS-area variability from a single lead, which is the basis of AVCI.

### 2.3 ML approaches - ECG interpretation

ML is applied to classify heart conditions based on ECG data. Used a bidirectional gated recurrent unit with a bottleneck attention mechanism to detect congestive heart failure (CHF) on the BIDMC and CHF-RR-interval databases, achieving 99.1% accuracy (9). Accurate CHF detection by converting one-dimensional ECG data into an Electrocardiomatrix (10). Worked on the model for predicting arrhythmia and ischemia, making it easier to interpret (15), and traditional machine-learning methods with engineered features still perform well for heartbeat classification (16, 17). Convolutional and hybrid neural networks have been used to distinguish normal rhythm (18, 20) and to enable lightweight Internet of Things applications (21). Although these methods are accurate, they usually require large, labeled datasets and often give results that are hard to interpret physiologically. By contrast, AVCI provides a single, interpretable value that is based directly on the signal and its underlying mechanism.

### 2.4 Ventricular repolarisation and morphology markers

Another research area focuses on markers from ventricular depolarization and repolarization. Changes in a person's QT-interval are linked to future heart problems and death in a large long-term study (22). QRS, QTc, and JTc intervals were associated with the risk of sudden cardiac death in the general population (23). DL approaches have been used to identify repolarization characteristics in congenital long-QT syndrome (24). To estimate the corrected QT interval, it was measured directly using DL approaches and various methods for extracting features from signals in different ways for further evaluation (25, 26). To understand how the human body regulates its temperature and dissipates excess heat. The QRS complex varies with anatomical differences (27). Most of these markers are based on measurability. Absolute interval lengths or shape templates, unlike AVCI, which employs beat-to-beat changes that use the QRS complex as the coupling signal rather than just the intervals (28).

### 2.5 Summary and identified gap

The literature review identifies an important gap in the methods currently used to analyze cardiac signals. HRV reflects fluctuations in the timing between beats and is a common method for evaluating autonomic nervous system activity. HRV, however, is not a reflection of changes in the electrical activity of the ventricles (as seen in the QRS complex). The existing coherence-based techniques generally rely on additional physiological signals, multiple ECG leads, or precise definitions of ECG features. These requirements make the system more complex and restrict its practical use. It is possible to enhance detection performance using ML methods, but these tend to be complex, require massive amounts of data, and offer little physiological interpretability. This makes the connection between autonomic regulation and modulation of ventricular electrical activity poorly understood. So far, there is no approach to quantify the spectral coherence between RR-interval variability and QRS-area variability with only one ECG lead. It's a definite research gap in autonomic–ventricular electrical morphology interaction. The present study addresses this gap by introducing AVCI, a simple, interpretable, single-lead ECG method that quantifies the coherence between RR-interval and QRS-area variability; this coherence is interpreted, as a physiological hypothesis, as a measure of autonomic–ventricular coupling.

## 3 Methodology

### 3.1 Proposed framework

This study presents AVCI, a new single-lead ECG method to measure coherence between autonomic and ventricular electrical dynamics. Raw ECG signals are filtered using a Butterworth band-pass filter between 0.5 and 40 Hz to remove noise. R-peaks are identified with an adaptive MAD-based threshold, and RR intervals are measured. The QRS area is calculated to represent the heart's electrical activity. From RR intervals and QRS area, two signals are created: V(t) for autonomic variability and C(t) for morphology variability. Both signals are normalized and resampled at 1 Hz. Welch spectral coherence analysis is then used to measure how well these signals are synchronized. The coherence values in the high-frequency range (0.15–0.40 Hz) are added to obtain the AVCI value. Finally, the coherence method is evaluated using an ML classifier and statistical analysis to assess its ability to differentiate healthy individuals from those with heart failure or arrhythmias as shown in Figure 1 and explained in Algorithm 1.

**Figure 1:**
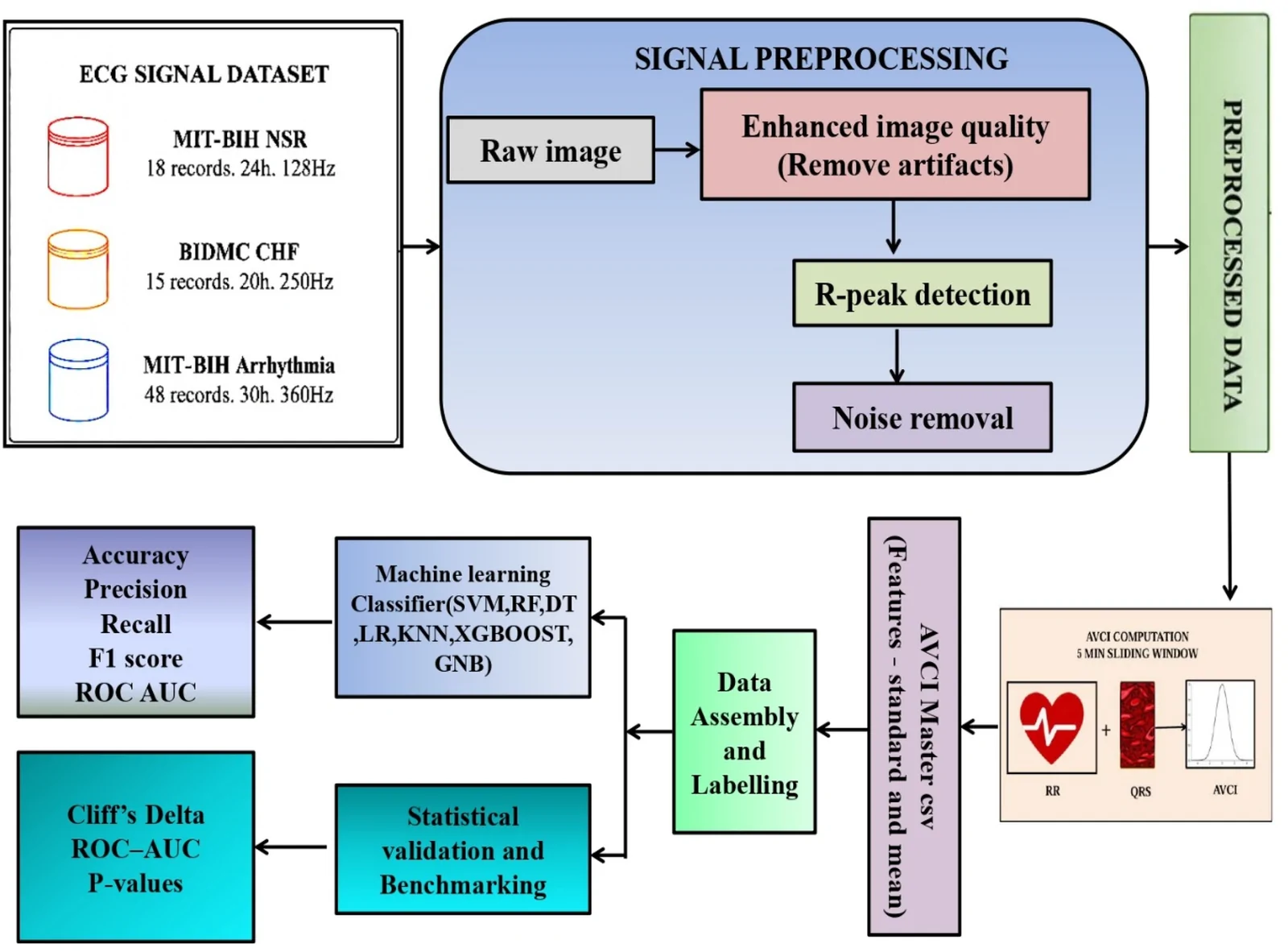
AVCI overall proposed framework.

Algorithm 1 **AVCI**

**1 Input**: Single-lead ECG, high-frequency autonomic band 0.15–0.40 Hz

**2 Output**: AVCI ∈ [0, 1], a scalar autonomic–ventricular coherence index (the greater, the better)

**3 Preprocessing:**

(a) Bandpass filter x(t) using a 3rd-order zero-phase (0.5 to 40 Hz) to give xf(t).

(b) Calculate envelope env(n) = | xf(n) |.

(c) Identify R-peaks env(n) > median(env) + 1.5 * MAD (env) with minimum separation of 0.25 * fs.

**4. Feature signals (i-th beat)**

(a) RR Interval: RR(i) = (ri−ri−1)/f s

(b) QRS area: QRS_area(i) = ∫ from (ri−60 ms) to (ri + 60 ms) of |xf(t)| dt

(c) Autonomic variability: V(i) = | RR(i)−RR(i−1) |

(d) Morphology variability: C(i) = | QRS_area(i)−QRS_area (i−1) |

**5 Normalization and resampling**

(a) Vnorm(i) = (V(i)−*μ*V)/*σ*V; Cnorm(i) = (C(i)−μC)/*σ*C

(b) Resample Vnorm and Cnorm onto a uniform 1 Hz grid.

**6. Spectral coherences (Welch's method)**

Coh^2^(f) = | PVC(f) |^2^/[ PVV(f) · PCC(f)], 0 ≤ Coh^2^(f) ≤ 1

**7 Band-normalized integration**

AVCI = (1/(fH−fL)) · ∫ from fL to fH of Coh^2^(f) df

**8 Classification (detection)**

(a) Calculate AVCI for each recording.

(b) Use a threshold or classifier to differentiate healthy (NSR) from diseased.

(c) Calculate discrimination accuracy/AUC considering the true value.

**9 End**

### 3.2 Dataset

This study was conducted using PhysioNet databases from four different datasets containing a total of 88 records. Three datasets with 81 records are used for method development and fourth dataset with 7 records are used for preliminary independent validation. Table 1 shows the dataset details from the PhysioNet Databases.

**Table 1:** Native dataset details.

Database	Role	Records	Duration
MIT-BIH NSR	Development healthy	18	∼24h
BIDMC CHF	Development disease	15	∼20h
MIT-BIH Arrhythmia	Development diseases	48	∼30min
MIT-BIH LTDB	Preliminary independent validation	7	14–24h
Total		88	

The first MIT-BIH Normal Sinus Rhythm Database (NSR) (21, 29) has 18 subjects with no significant heart disease. Recordings are 24 h long at a sampling rate of 128 Hz (128 samples per second). This dataset serves as a baseline for a healthy control group and shows what a normal heart rhythm looks like over an extended period. Each subject gives us approximately 800–1,200 AVCI windows.

The second dataset serves as a disease group and is used to study how heart failure affects ECG patterns. BIDMC Congestive Heart Failure Database (CHFDB) (12, 14, 30) comprises 15 subjects with severe heart failure, specifically classified as New York Heart Association (NYHA) Class III–IV, indicating advanced disease. This means subjects have symptoms even at rest. It has long ECG recordings lasting 20 h at a sampling rate of 250 Hz. Compared to NSR, CHF patients often exhibit reduced heart rate variability, abnormal rhythms, and weakened cardiac signals. Therefore, this dataset represents a disease group (CHF).

The third MIT-BIH Arrhythmia Database (31–33) has a mixed population of individuals with and without arrhythmias. It includes 48 subjects with various heart rhythm disorders, and each was recorded for 30 min at a sampling rate of 360 Hz. Each recording has beat-by-beat annotations with labels such as N-normal, V-premature ventricular contraction, and f-atrial fibrillation beat. This follows subclassifications: NORMAL, PVC, AF, PAC, MIXED. The classification of records based on the proportion of abnormal beats includes atrial fibrillation (AF) if atrial fibrillation beats exceed 5%, PVC-dominant (Premature Ventricular Contractions) if ventricular beats exceed 10%, PAC-dominant (Premature Atrial Contractions) if atrial beats exceed 5%, and normal if abnormal beats are less than 2%. If there is more than one abnormality in a recording but none is predominant, it is a mixed recording. Labeling is performed at the beat level and linked to detailed beat-level annotations, which are useful for automated cardiovascular analyses and classification tasks.

The final and fourth dataset used for preliminary independent validation was the MIT-BIH Long-Term ECG Database (LTDB) (34). It is a long-duration (14–24 h) ambulatory ECG database sampled at 128 Hz from a cohort of cardiac monitoring patients. It tests AVCI's ability to generalize to data it wasn't trained on and recorded under conditions like those in the NSR database.

### 3.3 Data preparation

The collected dataset was organized for analysis under native, harmonized, and Duration-Stratified Two-Tier Analysis because the databases differed in sampling frequency, duration, and acquisition. The original configurations were preserved in native analysis. An upsampled analysis was performed at 360 Hz for 30 min to distinguish sampling-rate from duration effects. A harmonized analysis used a common 128 Hz and 30 min. As the observed duration in the recording signals was varying, Duration-Stratified Two-Tier Analysis was conducted with Tier 1 (long duration, ∼19 h, NSR vs. CHF) and Tier 2 (short duration, 30 min, NSR vs. MIT-BIH Arrhythmia). Hence, these versions of the datasets are used in the further experimental studies.

### 3.4 ECG signal pre-processing and acquisition standardization

Signal pre-processing plays a vital role in preparing ECG signals for further analysis. ECG signals often carry common disturbances during the recording of electrical activity, such as baseline wandering, instrumental noise, and electrode motion artifacts. These unwanted components affect the signal characteristics and reduce the accuracy of the automated analysis system. Since the collected datasets, 128 Hz for MIT-BIH NSR, 250 Hz for BIDMC CHF, and 360 Hz for MIT-BIH Arrhythmia, carry different sampling frequencies and contain noises, the pre-processing steps, including noise removal and R-peak detection which are explained below as shown in Figure 2.

**Figure 2:**
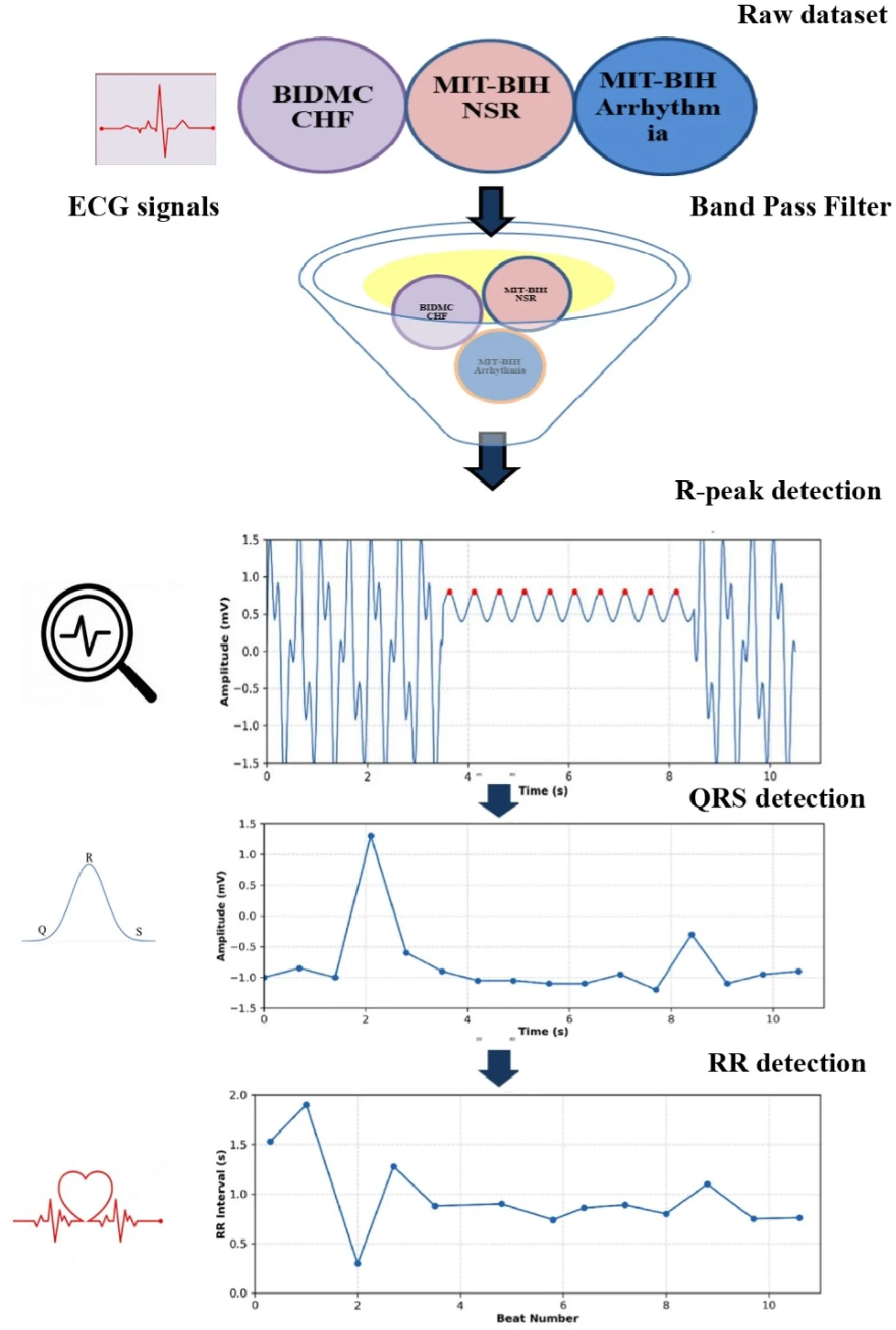
ECG signal preprocessing pipeline.

#### 3.4.1 Bandpass filtering

A 3rd-order Butterworth bandpass filter (16, 33, 35) (0.5–40 Hz) was used on the raw ECG signal. Frequencies below 0.5 Hz are mostly slow baseline drift, while those above 40 Hz are usually electrical or muscle noise. Butterworth filter was chosen for its flat passband with normalized lower and upper cut-off frequencies for the desired passband, as shown in Equations (1) and (2). To avoid phase distortion, zero-phase filtering was used to filter the signal forward and backward (filtfilt). The Butterworth frequency response is shown in Equation (3).

The cut-off frequencies were normalized using the Nyquist frequency (36) defined as:

$$f_{\mathrm{nyq}}=\frac{\;f_{s}}{2}$$ (1)

where $f_{s}\;$ denotes the sampling frequency and $f_{\mathrm{nyq}\;}$ represents the Nyquist frequency.

The normalized cut-off frequencies were computed using:

$$W_{L}=\frac{\;f_{L}}{\;f_{\mathrm{nyq}}},W_{H}=\frac{\;f_{H}}{\;f_{\mathrm{nyq}}}$$ (2)

where $f_{L}$ and $f_{H}$ correspond to the lower and upper cutoff frequencies, respectively.

The Butterworth frequency response equation:

$$|H(\;j\omega ){|}^{2}=\frac{1}{1+{\left(\frac{\omega }{\omega_{c}}\right)}^{2n}}$$ (3)

where H(jω) -represents the filter frequency response, $\omega_{c}$ -denotes the cut-off frequency, and n - the order of the Butterworth filter.

#### 3.4.2 R-peak detection

The R-peak is the highest positive point in the QRS complex of an ECG signal and shows ventricular depolarisation. Because it stands out most in the ECG waveform, it is used to detect heartbeats, identify the QRS complex, and calculate RR intervals.

To find R-peaks, the amplitude envelope of the filtered ECG signal was first calculated by taking its absolute values, as shown in Equation (4) (37):

$$\mathrm{env}=|\mathrm{EC}G_{\mathrm{filtered}}(n)|$$ (4)

Next, an adaptive threshold is calculated using both the median and the MAD of the envelope signal. This method reliably detects R-peaks, even in the presence of baseline changes or noise, as shown in Equations (5) and (6).

Threshold=median(env)+1.5×MAD(env) (5)

where

$$\mathrm{MAD}\;(x)=\mathrm{median}\;(|x_{i}-\mathrm{median}\;(x)|)$$ (6)

MAD reliably measures variability in the data. It is less sensitive to outliers than the standard deviation, making it suitable for ECG signals with irregular noise spikes.

R-peaks were detected by using the find_peaks function with an adaptive threshold and a minimum peak distance set to match a 250 ms refractory period. The detection rule is given by Equation (7), derived from Equations (4–6).

$$R=\{n_{k}\mathrm{env}(n_{k})>\mathrm{Threshold},n_{k}-n_{k-1}\geq 0.25f_{s}\}$$ (7)

Here $f_{s}\;$ is the sampling frequency, and $n_{k}$ is the sample index of the detected R-peak. The peak locations were identified by using the find_peaks () function with these parameters:

$$\begin{array}{cc} \mathrm{Height}=\mathrm{Threshold}\;\mathrm{where}\;\mathrm{distance}=0.25 & \;f_{s} \end{array}$$

#### 3.4.3 R-peak detection validation

The accuracy of R-peak recognition was validated using expert-verified annotations from the MIT-BIH Normal Sinus Rhythm (NSR) and Arrhythmia databases (66 records). Because PhysioNet records its annotations as unaudited automatic output rather than as an expert-verified reference, the BIDMC-CHF database was left out of this validation. The R-peak detection yielded an F1-score of 0.892, a sensitivity of 0.973, and a positive predictive value (PPV) of 0.836 with a ± 50 ms matching tolerance. Two extremely noisy arrhythmia recordings (107 and 217) explained most of the variability across recordings; removing them raised the F1-score to 0.910. However, the same algorithm was used for both R-peak identification and AVCI computation for the CHF database, enabling consistent AVCI results for all three datasets.

#### 3.4.4 QRS-window sensitivity

To assess sensitivity to the fixed 120-ms window, AVCI was calculated using QRS-area windows of 80, 120, 150, and 180 ms under both native and harmonized conditions, as explained in Section 4.9.

### 3.5 AVCI computation

The AVCI measures the degree of association between beat-to-beat variations in the RR interval and variations in the QRS complex area. This analysis focuses primarily on the high-frequency range of the RR interval (0.15 to 0.40 Hz), as shown in Figure 3.

**Figure 3:**
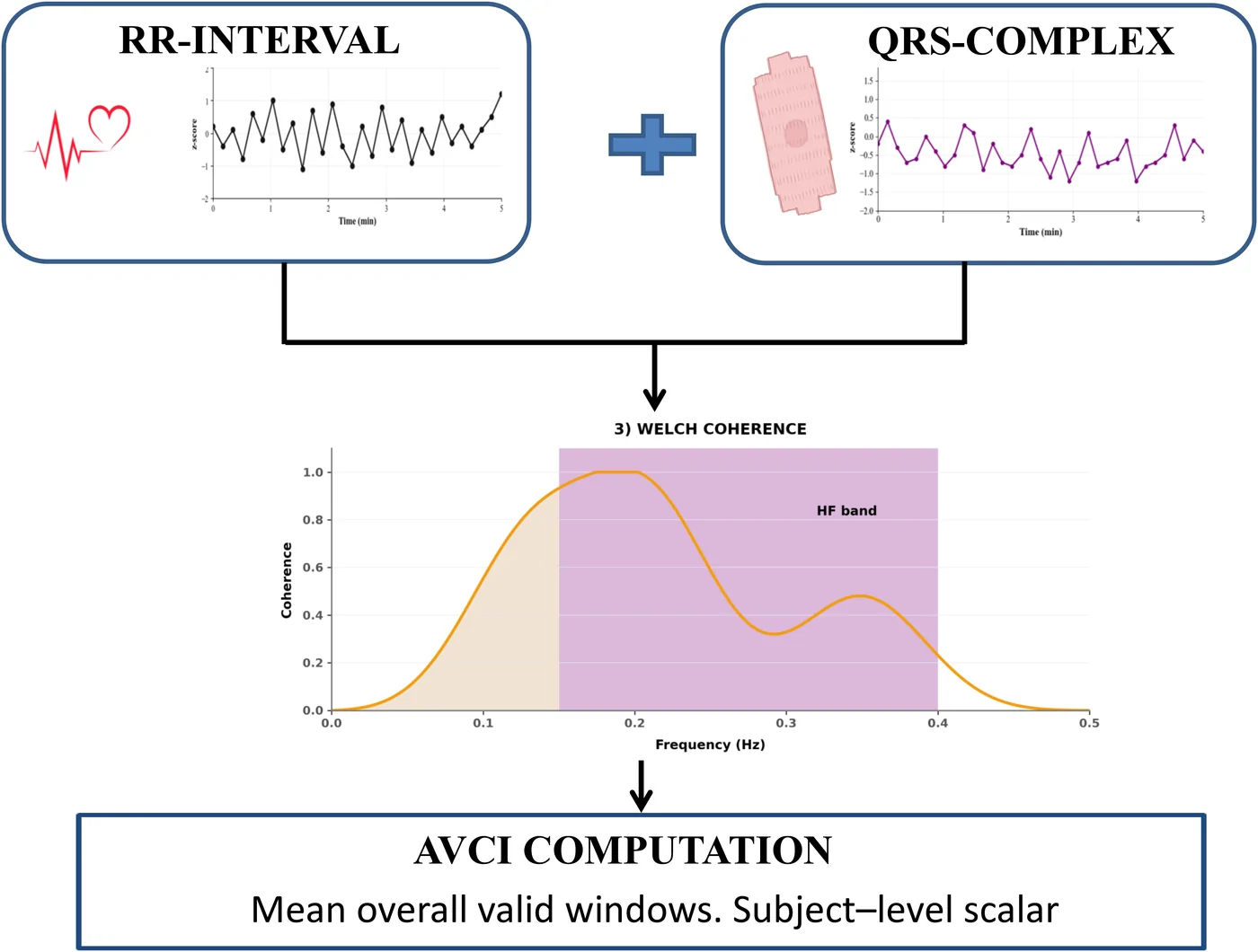
Overall AVCI computation pipeline.

Per-beat QRS area was computed as the trapezoidal integral of the absolute filtered ECG over a 120 ms window centered symmetrically on each R-peak. This morphology feature is robust to variation in sampling rate and requires no delineation of the T-wave, Q-wave, or S-wave, distinguishing it from interval-based and intra-QT approaches (14, 27).

#### 3.5.1 Feature extraction

The detected R-peaks were used to extract two types of features: autonomic (RR interval) and ventricular cardiac dynamics (QRS area). These former features are used to build the autonomic signal C(t) and the ventricular signal V(t), which are then used for subsequent AVCI.

##### 3.5.1.1 Features

(i) QRS Area Computation

For each detected R-peak, a fixed 120-ms window centered on the R-peak (60 ms before and 60 ms after) was extracted from the filtered ECG signal, as shown in Equation (8). The QRS area was then calculated as the area under the absolute value of the ECG waveform within this window (38–43):

$$\mathrm{QR}S_{\mathrm{area}}(i)=\overset{r_{i}+60\mathrm{ms}}{\underset{r_{i}-60\mathrm{ms}}{\int }}|\mathrm{EC}G_{\mathrm{filtered}}(t)|\;\mathrm{dt}$$ (8)

where $r_{i}$ denotes the location of the $i^{th}$ R-peak.

Using the trapezoidal rule in Equation (9), the integral was mathematically approximated:

$$\mathrm{QR}S_{\mathrm{area}}(i)\approx \mathrm{trapz}(|\mathrm{EC}G_{\mathrm{filtered}}[r_{i}-\mathrm{half}:r_{i}+\mathrm{half}]|)$$ (9)

This QRS area is a morphological representation of the entire ventricular depolarisation activity linked to each heartbeat.

(ii) RR Interval Computation

The time difference between successively observed R peaks was used to calculate RR intervals shown in Equation (10), which were then converted to milliseconds:

$$\mathrm{RR}(i)=\frac{R_{i+1}-R_{i}}{\;f_{s}}\times 1000$$ (10)

Where $f_{s}$ is the sampling frequency, and *R_i* and *R*_(*i* + 1) indicate successive *R*-peak sample positions.

The RR interval forms the basis for heart rate variability (HRV) analysis and provides information about beat-to-beat changes in cardiac rhythm. To reduce the influence of false R-peak detections and physiologically unrealistic heart rates, RR intervals outside the range of 400–1,500 ms were excluded. This range corresponds approximately to heart rates between 40 and 150 beats per minute.

#### 3.5.2 Signals from features

(i) Autonomic Variability Signal V(t)

The autonomic variability signal V(t) represents temporal variations in cardiac rhythm associated with autonomic nervous system activity. It is typically derived from the beat-to-beat RR interval fluctuations (Equation (10)) and is used to analyze heart rate variability and cardiovascular regulation.

The autonomic variability signal was defined as the absolute beat-to-beat change in RR intervals shown in Equation (11):

V(i)=∣RR(i)−RR(i−1)∣ (11)

where RR(i) represents the $i^{\mathrm{th}}$ RR interval in milliseconds.

Larger values of V(i) Indicate stronger short-term modulation of heart rate by the autonomic nervous system.

(ii) Ventricular electrical morphology variability signal C(t)

The ventricular electrical morphology variability signal C(t) represents temporal variations in ECG waveform morphology associated with cardiac electrical activity. It is derived from beat-to-beat changes in the QRS (Equation (9)) area of the ECG waveform. It is used to analyze structural and functional abnormalities in cardiac conduction and myocardial activity, as explained in Equation (12).

The ventricular electrical morphology variability signal was defined as the absolute beat-to-beat change in QRS area:

$$C(i)=|\mathrm{QR}S_{\mathrm{area}}(i)-\mathrm{QR}S_{\mathrm{area}}(i-1)|$$ (12)

where $\mathrm{QR}S_{\mathrm{area}}(i)$ denotes the QRS area associated with the $i^{\mathrm{th}}$ heartbeat.

Elevated values of C(i) reflect increased beat-to-beat variability in ventricular depolarisation morphology, potentially associated with changes in conduction dynamics, stroke volume, or ectopic activity.

#### 3.5.3 Z-score normalization

To ensure consistent scaling and reduce the influence of amplitude variations, both the autonomic variability signal V(t) and ventricular electrical morphology variability signal C(t) were standardized using z-score normalization Equations (13 and 14):

$$V_{\mathrm{norm}}(i)=\frac{V(i)-\mu_{V}}{\sigma_{V}}$$ (13)

$$C_{\mathrm{norm}}(i)=\frac{C(i)-\mu_{C}}{\sigma_{C}}$$ (14)

where μ and σ represent the mean and standard deviation of the corresponding signal, respectively. Normalization ensures comparable scaling across signals and prevents magnitude differences during coherence analysis.

#### 3.5.4 Resampling to a uniform time grid

Normally, our heartbeats occur at irregular intervals. The derived signals were similarly irregular and were resampled onto a uniformly sampled temporal grid using linear interpolation. Beat occurrence times were computed as shown in Equations (15 and 16):

$$t_{\mathrm{beats}}=\frac{\mathrm{peaks}[1:]}{\;f_{s}}$$ (15)

where peaks denotes the detected R-peak locations and $f_{s}$ is the sampling frequency.

A uniformly sampled time vector with a sampling rate of 1 Hz was then generated as:

$$t_{\mathrm{uniform}}=[t_{\min},t_{\min}+1,t_{\min}+2,\ldots ,t_{\max}]$$ (16)

The normalized variability signals were subsequently interpolated onto this uniform time grid to enable spectral analysis using evenly sampled data.

#### 3.5.5 Spectral coherence estimation

Spectral coherence between the normalized autonomic variability signal V(t) and ventricular electrical morphology variability signal C(t) was estimated using Welch's method with a sampling frequency of 1. The magnitude-squared coherence was defined in Equation 17:

$$\mathrm{Co}.:(f)=\frac{|P_{\mathrm{VC}}(f){|}^{2}}{P_{\mathrm{VV}}(f)P_{\mathrm{CC}}(f)}$$ (17)

where $P_{VC}(f)$ represents the cross-spectral density between V(t) and C(t), $P_{\mathrm{VV}}(f)$ represents the power spectral density of V(t), $P_{\mathrm{CC}}(f)$ represents the power spectral density of C(t).

Equation (18) describes the coherence spectrum, which satisfies:

$$0\leq \mathrm{Co}h^{2}(f)\leq 1$$ (18)

where values close to 0 indicate weak linear coupling and values close to 1 indicate strong coupling between the two signals at the frequency *f*.

Normal human breathing rate is about 9–24 breaths per minute. This frequency band was chosen as 0.15–0.40 Hz because it includes normal respiratory indices and covers the range of frequencies associated with respiratory sinus arrhythmia (RSA), which reflects the autonomic modulation of cardiac activity. This autonomic nervous system modulation of heart rate (HR) by the respiratory cycle is called RSA. A higher AVCI in this frequency band will represent, a higher AVCI will represent greater autonomic–cardiac coupling.

#### 3.5.6 AVCI calculation

##### 3.5.6.1 AVCI for single window

For each sliding analysis window, the AVCI was computed as the normalized area under the coherence spectrum within the HF band, mathematically explained in Equation (19):

$$\mathrm{ACCI}=\frac{1}{0.25}\overset{0.40}{\underset{0.15}{\int }}\;{\mathrm{Coh}}_{\mathrm{VC}}^{2}(f)\;\mathrm{df}$$ (19)

The integral was numerically approximated using the trapezoidal rule. Since the coherence spectrum is bounded between 0 and 1, the AVCI value also satisfies:

0≤AVCI≤1

Values approaching 1 indicate strong autonomic–ventricular electrical morphology coupling within the respiratory frequency range, while lower values indicate weaker physiological coupling, as shown in Figure 4.

**Figure 4:**
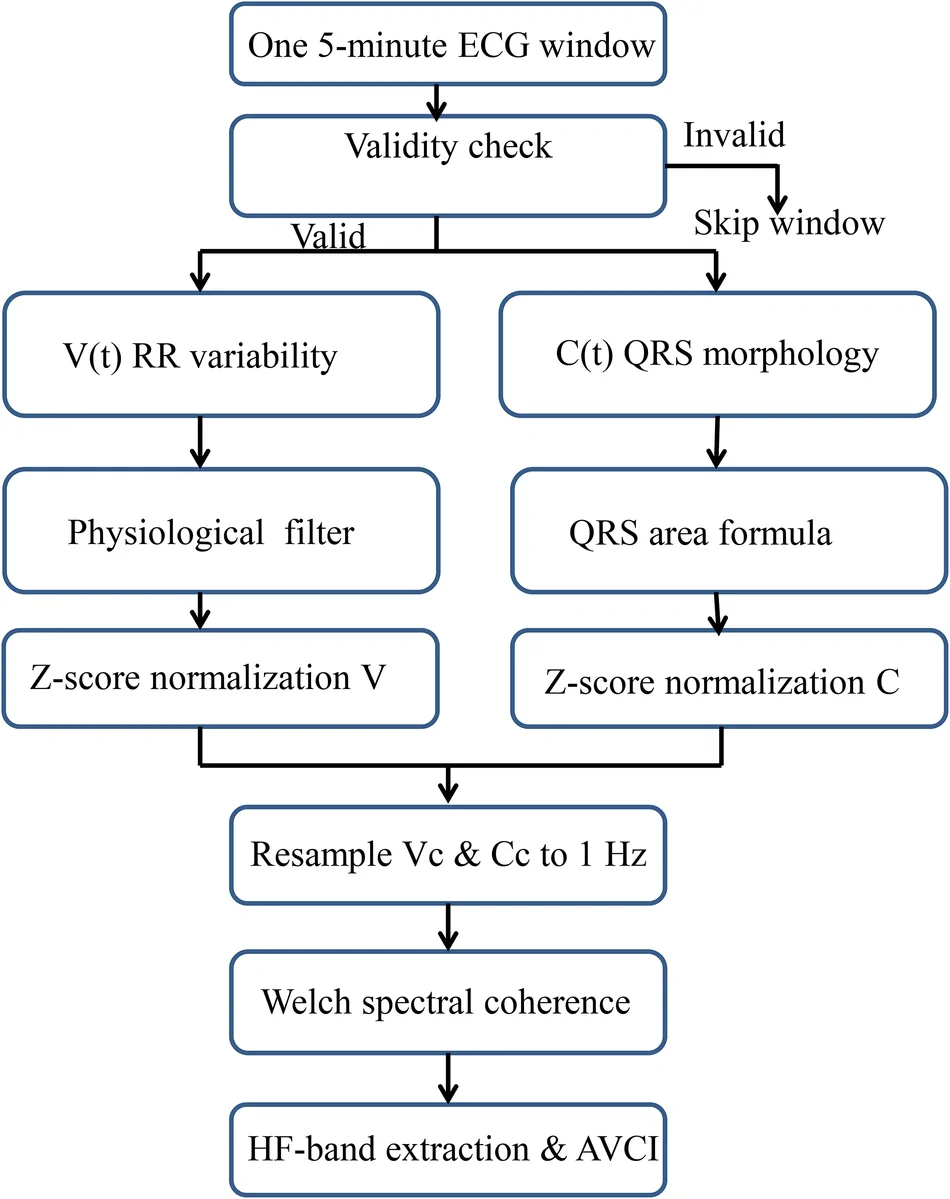
Single-window AVCI computation.

##### 3.5.6.2 Subject-level AVCI

AVCI was computed using a 5-minute sliding window with a 1-minute step size, as shown in Figure 5. Within each window, two beat-indexed variability signals were constructed, and their spectral coherence was evaluated.

**Figure 5:**
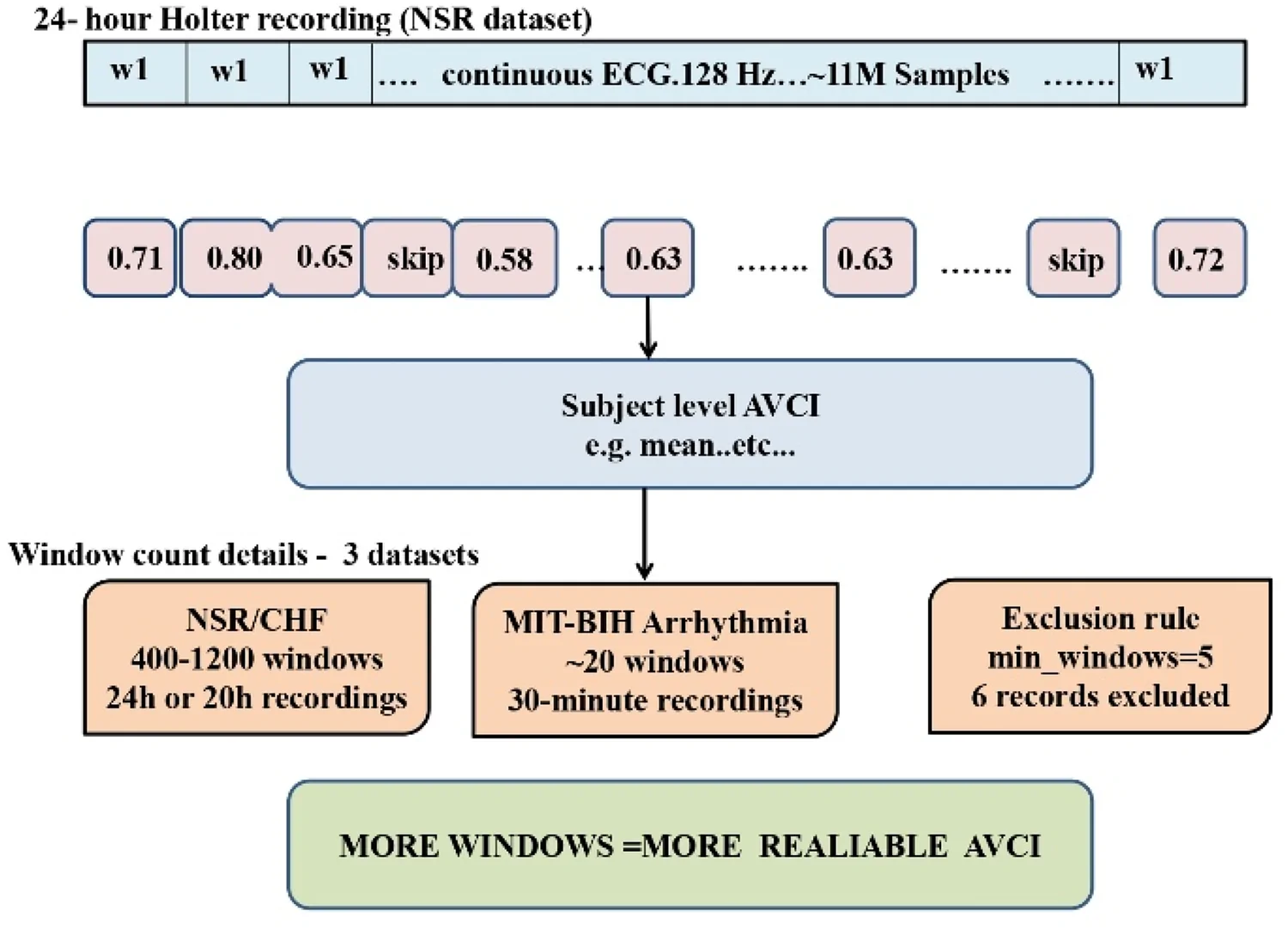
Subject-level AVCI computation.

This is done for every valid 5-minute window in the recording (a sliding window with a 1-minute step), and the mean is taken to reduce any transient noise and produce a stable per-subject scalar. AVCI_subject = mean ([AVCI_w1, AVCI_w2, .., AVCI_wN]) Less than 30 beats or less than 60s grid, excluded (only windows with ≥30 beats and ≥60s grid included).

### 3.6 Dataset assembly and labeling

The originally extracted dataset was labeled according to three rhythm classes: NSR, CHF, and ARR. The extracted features (AVCI mean and standard deviation) are stored in the master CSV file. For machine-learning classification, these three classes were converted into two classes to evaluate AVCI's ability to distinguish NSR from non-NSR recordings. NSR was labeled as class 1, whereas CHF and ARR were combined as non-NSR and labeled as class 0.

### 3.7 ML classifier

Eight ML classifiers were used, including Logistic Regression, Decision Tree, Random Forest, Support Vector Machine (SVM), Gradient Boosting, K-Nearest Neighbors (KNN), Gaussian Naïve Bayes, and XGBoost. Two datasets were examined (the native one and the harmonized one). For each dataset, both the naturally occurring class distribution and a balanced version with equal numbers of NSR and non-NSR recordings were evaluated. The balanced dataset included 36 recordings: 18 NSR and 18 non-NSR. Model performance was evaluated independently on the original and balanced datasets using repeated stratified five-fold cross-validation. Five-fold cross-validation was repeated 20 times, resulting in 100 validation folds for each classifier within each dataset. Stratification maintained the class distribution between the training and validation subsets. For each fold, the classifier was trained on the training data and evaluated on the held-out validation data. Without hyperparameter tuning, the models were trained with predetermined hyperparameters.

Classifier performance was assessed using accuracy, precision, sensitivity (recall), specificity, F1-score, balanced accuracy, and ROC-AUC. Accuracy represented the proportion of correctly classified recordings. Sensitivity represented the proportion of NSR recordings correctly identified as NSR, while specificity represented the proportion of non-NSR recordings correctly identified as non-NSR. Balanced accuracy was calculated as the mean of sensitivity and specificity. F1-score represented the harmonic mean of precision and sensitivity.

### 3.8 AVCI differences between groups

AVCI reflects physiological changes in disease. There are three distinct mechanisms:

#### 3.8.1 Healthy hearts (high AVCI)

Breathing drives both V(t) and C(t) through a shared pathway of the vagus nerve, creating RSA (RR variation at breathing frequency), and the same respiratory cycle changes ventricular preload via intrathoracic pressure (Frank-Starling mechanism → QRS area variation). Both oscillate at the same frequency → high coherence → high AVCI.

#### 3.8.2 CHF (lower AVCI)

Heart failure causes sympathetic overdrive and vagal withdrawal. This flattens RSA — the HF component of RR variability shrinks. At the same time, ventricular remodeling makes QRS morphology less responsive to autonomic input. Both mechanisms reduce the coupling between V and C at HF frequencies.

#### 3.8.3 PVC-dominant arrhythmia (lowest AVCI)

Premature Ventricular Contractions are beats that originate in the ventricle rather than the SA node. They reset ventricular activation irregularly and independently of autonomic modulation. Every PVC inserts a large random perturbation into C(t) that has no corresponding perturbation in V(t) → directly destroys HF coherence → very low AVCI.

### 3.9 Benchmark HRV metrics

The standard HRV metrics SDNN, RMSSD, and mean heart rate were calculated from the entire recording for comparison with NSR and CHF. The HRV benchmark was limited to NSR and CHF subjects because standard time-domain HRV measures are confounded by ectopic beats in arrhythmia recordings, where frequent premature contractions produce artificially high successive difference measures that are not related to autonomic tone. Within-window z-score normalization and the physiological RR-interval filter make AVCI relatively immune to this confounding.

### 3.10 Statistical analysis

The Kruskal–Wallis H-test was used to assess differences between groups when data were available for 3 or more groups. Pairwise comparisons between NSR and each disease group used two-sided Mann–Whitney U tests with Bonferroni correction for six comparisons. Effect sizes were quantified using Cliff's delta, interpreted as negligible (|*δ*|<0.147), small (0.147–0.330), medium (0.330–0.474), or large (>0.474). Diagnostic performance was described using ROC-AUC, and 95% CIs were estimated from 2,000 bootstrap resamples. The analyses were carried out in Python 3.11 with the SciPy, scikit-learn, and WFDB packages.

## 4 Results

The experiment is conducted using a co-core GPU. The Python libraries used to implement the proposed architecture are NumPy, pandas, SciPy, wfdb, scikit-learn, xgboost, and matplotlib. The code for the proposed AVCI method and its extraction from the native dataset is made available in a GitHub repository: https://github.com/JayaSreejai/AVCI.git.

### 4.1 AVCI distribution by group

Table 2 shows that the descriptive distribution of AVCI was different under native recording conditions among the cardiac groups. The MIT-BIH NSR group exhibited the highest mean AVCI (0.663 ± 0.105), compared with 0.526 ± 0.095 for BIDMC CHF, 0.526 ± 0.146 for ARR-NORMAL, 0.506 ± 0.092 for PVC-dominant, and 0.531 ± 0.133 for MIXED recordings. The mean AVCI values for the two subjects in AF and PAC recordings were 0.487 ± 0.030 and 0.360 ± 0.030, respectively. The two subgroups were described and excluded from inferential analyses and effect size estimation. Following Table 3 shows harmonization to 30 min at 128 Hz, the mean AVCI values were 0.5855 ± 0.1857 for NSR, 0.4937 ± 0.1008 for CHF, 0.5373 ± 0.1270 for ARR-NORMAL, 0.4950 ± 0.0889 for PVC-dominant, and 0.5315 ± 0.1398 for MIXED recordings. The corresponding AF and PAC means were 0.4976 ± 0.0008 and 0.3780 ± 0.0238, respectively. Under harmonization to 360 Hz for 30 min as shown in Table 4, the corresponding mean AVCI values were 0.5805 ± 0.1885, 0.4993 ± 0.1008, 0.5253 ± 0.1270, 0.5057 ± 0.0889, and 0.5312 ± 0.1398 for NSR, CHF, ARR-NORMAL, PVC-dominant, and MIXED groups, respectively. AF and PAC had mean values of 0.4873 ± 0.0140 and 0.3599 ± 0.0295, respectively. AVCI values vary among cardiac groups, with NSR showing the most coherence and PAC the lowest, as seen in Figures 6–8. NSR had a higher mean AVCI (0.6616) than CHF (0.5201) in Tier 1 utilizing the harmonized 19-hour recordings. The 30-minute analysis in Tier 2 revealed mean AVCI values of 0.5125 for arrhythmia recordings and 0.5835 for NSR. These findings show that AVCI values were consistently higher in NSR, with longer records showing a clearer distinction. This proves AVCI differences may reflect both disease status and acquisition characteristics. Since AVCI is sensitive to sampling rate and recording duration, signals were harmonized across databases to minimize these effects. Across all harmonization tiers, NSR consistently demonstrated higher AVCI values than CHF and arrhythmia groups, indicating that the NSR higher AVCI pattern is robustly disease-related while also sensitive to database and acquisition characteristics.

**Figure 6:**
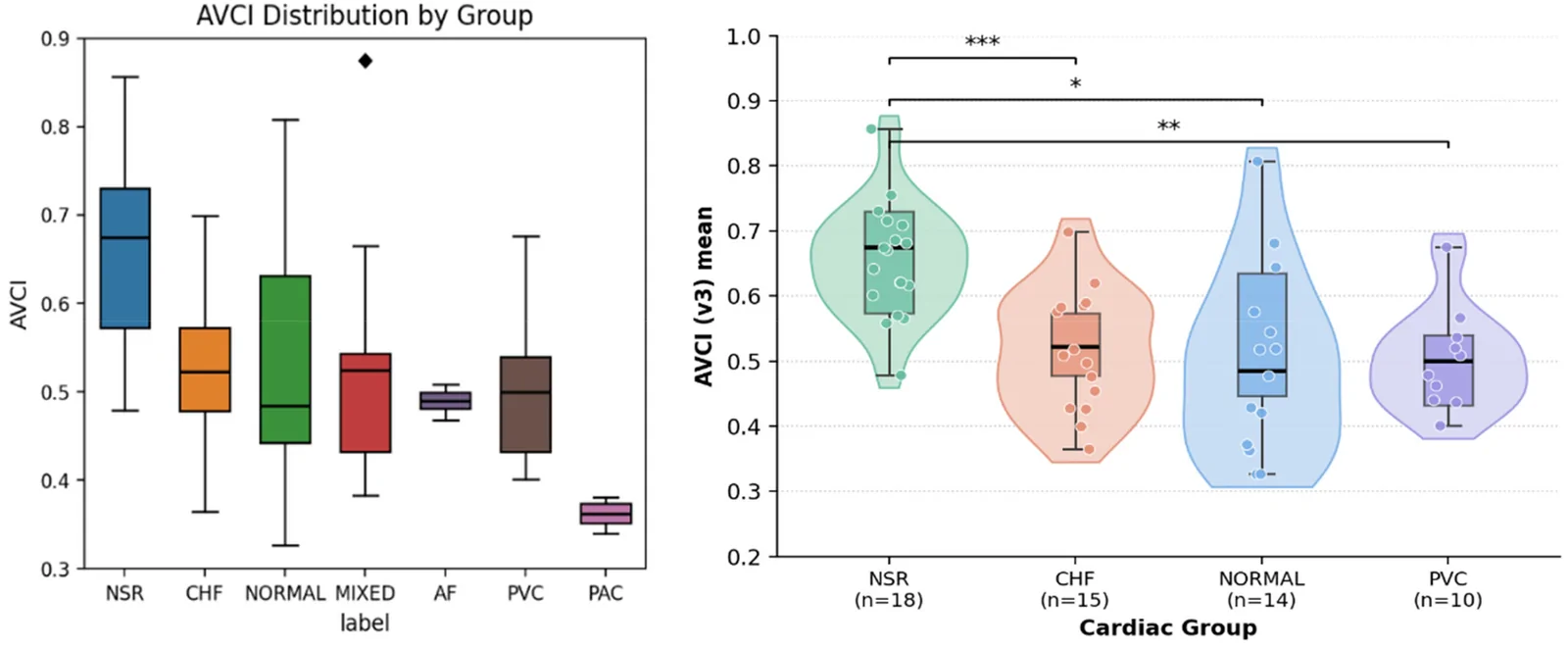
Violin and box plot of AVCI distribution across NSR, CHF, ARR-NORMAL, and PVC groups.

**Figure 7:**
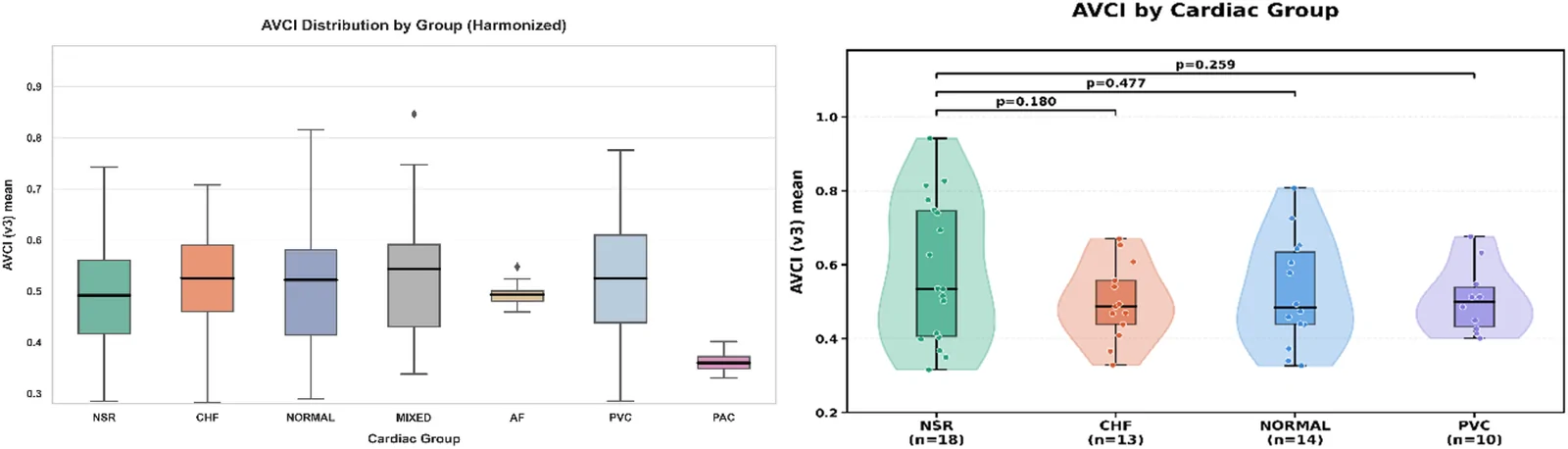
Violin and box plot of AVCI distribution across NSR, CHF, ARR-NORMAL, and PVC groups (harmonized 128 HZ, 30 min).

**Figure 8:**
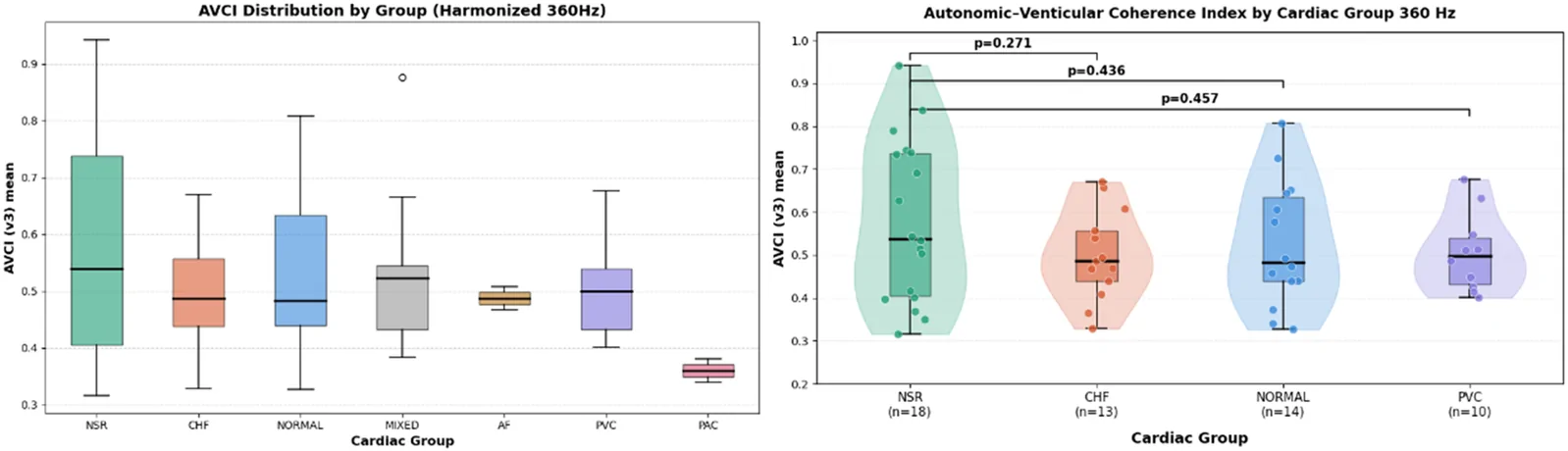
Violin and box plot of AVCI distribution across NSR, CHF, ARR-NORMAL, and PVC groups (harmonized 360 HZ, 30 min).

**Table 2:** AVCI descriptive statistics by cardiac group (native database).

Group		*n*	Mean AVCI	SD	Min	Max	Recording
MIT-BIH NSR		18	0.663	0.105	0.478	0.856	∼24 h, 128 Hz
BIDMC CHF		15	0.526	0.095	0.365	0.698	∼20 h, 250 Hz
MIT-BIH	ARR-NORMAL	14	0.526	0.146	0.327	0.808	∼30 min, 360 Hz
	PVC-dominant	10	0.506	0.092	0.401	0.676	∼30 min, 360 Hz
	MIXED	13	0.531	0.133	0.383	0.877	∼30 min, 360 Hz
	AF	2	0.487	0.030	0.466	0.508	∼30 min, 360 Hz
	PAC	2	0.360	0.030	0.339	0.381	∼30 min, 360 Hz

**Table 3:** AVCI descriptive statistics by cardiac group (harmonized 128 HZ, 30 min).

Group		*n*	Mean AVCI	SD	Min	Max	Recording
MIT-BIH NSR		17	0.5855	0.1857	0.3126	0.4982	∼30 min, 128 Hz
BIDMC CHF		13	0.4937	0.1008	0.3514	0.3514	∼30 min, 128 Hz
MIT-BIH	ARR-NORMAL	13	0.5373	0.1270	0.3460	0.7990	∼30 min, 128 Hz
	PVC-dominant	10	0.4950	0.0889	0.3997	0.6602	∼30 min, 128 Hz
	MIXED	13	0.5315	0.1398	0.3722	0.8588	∼30 min, 128 Hz
	AF	2	0.4976	0.0008	0.4970	0.4982	∼30 min, 128 Hz
	PAC	2	0.3780	0.0238	0.3612	0.3948	∼30 min, 128 Hz

**Table 4:** AVCI descriptive statistics by cardiac group (harmonized 360 HZ, 30 min).

Group		*n*	Mean AVCI	SD	Min	Max	Recording
MIT-BIH NSR		18	0.5805	0.1885	0.3161	0.9418	∼30 min, 360 Hz
BIDMC CHF		13	0.4993	0.1008	0.3285	0.6701	∼30 min, 360 Hz
MIT-BIH	ARR-NORMAL	14	0.5253	0.1270	0.3265	0.8076	∼30 min, 360 Hz
	PVC-dominant	10	0.5057	0.0889	0.4008	0.6760	∼30 min, 360 Hz
	MIXED	13	0.5312	0.1398	0.3722	0.8768	∼30 min, 360 Hz
	AF	2	0.4873	0.0140	0.4663	0.5083	∼30 min, 360 Hz
	PAC	2	0.3599	0.0295	0.3390	0.3807	∼30 min, 360 Hz

### 4.2 Statistical group differences

The Kruskal–Wallis test showed a significant difference between groups under native recording conditions (H = 17.14, *p* = 0.0018). Pairwise Mann–Whitney U testing with Bonferroni correction showed higher AVCI values for NSR than CHF (U = 228, adjusted *p* = 0.005, Cliff's *δ* = 0.689), PVC-dominant recordings (U = 159, adjusted *p* = 0.006, Cliff's *δ* = 0.767), and MIXED recordings (U = 196, adjusted *p* = 0.010, Cliff's *δ* = 0.675). The comparison between NSR and ARR-NORMAL was not statistically significant (U = 190, adjusted *p* = 0.095), with a reported Cliff's *δ* of 0.508. However, NSR was significantly different from all disease records under native conditions (U = 844, adjusted *p* < 0.0001, Cliff's *δ* = 0.675). None of the NSR-disease comparisons following Bonferroni correction were statistically significant after harmonization to 128 Hz and 30 min. The NSR-versus-all-disease comparison yielded U = 558, raw *p* = 0.14279, adjusted *p* = 1.0000, and Cliff's *δ* = 0.239. When compared with CHF, ARR-NORMAL, PVC-dominant, and MIXED groups, the adjusted *p*-values were 1.0000, and Cliff's *δ* values were 0.294, 0.158, 0.271, and 0.149, respectively. Similarly, after harmonization to 360 Hz and 30 min, the NSR was not statistically significantly different from any disease (U = 580, raw *p* = 0.2240, adjusted *p* = 1.0000, Cliff's *δ* = 0.193). Comparisons with the CHF, ARR-NORMAL, PVC-dominant, and MIXED groups also had adjusted *p* = 1.0000, with Cliff's *δ* values of 0.167, 0.167, 0.178, and 0.085, respectively, as shown in Tables 5–7. NSR and CHF differed significantly, according to Tier 1 analysis (*p* = 0.000825, AUC = 0.844). The Tier 2 snapshot analysis, on the other hand, did not demonstrate statistical significance (*p* = 0.28091). This demonstrates how recording duration affects discriminating strength, with longer windows producing higher statistical separation.

**Table 5:** Pairwise Mann–Whitney U tests with Bonferroni correction (native database).

Comparison	*n* (dis.)	U	*p* (raw)	*p* (adj.)	Sig.	Cliff's *δ* (effect)
NSR vs. ALL disease	56	844	<0.0001	<0.0001	****	0.675
NSR vs. CHF	15	228	0.0008	0.005	**	0.689
NSR vs. ARR-NORMAL	14	190	0.0159	0.095	Ns	0.508
NSR vs. PVC	10	159	0.0010	0.006	**	0.767
NSR vs. MIXED	13	196	0.0017	0.010	*	0.675

**Table 6:** Pairwise Mann–Whitney U tests with Bonferroni correction (harmonized, 128 HZ, 30 min).

Comparison	*n* (dis.)	U	*p* (raw)	*p* (adj.)	Sig.	Cliff's δ (effect)
NSR vs. ALL disease	53	558	0.14279	1.0000	ns	0.239
NSR vs. CHF	13	143	0.1805	1.0000	ns	0.294
NSR vs. ARR-NORMAL	13	128	0.4768	1.0000	ns	0.158
NSR vs. PVC	10	108	0.2586	1.0000	ns	0.271
NSR vs. MIXED	13	127	0.5031	1.0000	ns	0.149

**Table 7:** Pairwise Mann–Whitney U tests with Bonferroni correction (harmonized, 360 HZ, 30 min).

Comparison	*n* (dis.)	U	*p* (raw)	*p* (adj.)	Sig.	Cliff's δ (effect)
NSR vs. ALL disease	54	580	0.2240	1.0000	ns	0.193
NSR vs. CHF	14	147	0.4361	1.0000	ns	0.167
NSR vs. ARR-NORMAL	14	147	0.4361	1.0000	ns	0.167
NSR vs. PVC	10	106	0.4574	1.0000	ns	0.178
NSR vs. MIXED	13	127	0.7037	1.0000	ns	0.085

Significance: ****p < 0.0001; **p < 0.01; *p < 0.05; ns, not significant. Global Kruskal–Wallis: H = 17.14, *p* = 0.0018. Group differences under harmonized settings are indicated in Section 4.9, where NSR-vs.-all-disease was not statistically significant (*p* = 0.14). These statistical variations represent native recording conditions.

### 4.3 Machine learning

Eight ML classifiers were used to classify NSR and non-NSR (diseased) recordings under native and harmonized acquisition conditions. In the native database condition before balancing, accuracy was between 70.4% and 81.0%, and ROC-AUC was between 76.5% and 86.3%. KNN and Gaussian Naïve Bayes achieved the highest accuracy (81.0%), while KNN achieved the highest ROC-AUC (86.3%). SVM's ROC-AUC value was 78.9%, and XGBoost's value was 83.7%, as shown in Table 8. After balancing, native database accuracy ranged from 71.3% to 79.5%, and ROC-AUC ranged from 76.5% to 89.4%. KNN accuracy was 79.5% and its ROC-AUC was 85.7%, whereas Gaussian Naïve Bayes and Logistic Regression both had ROC-AUC of 89.4%, as shown in Table 9. Table 10 shows that, after harmonizing to 128 Hz and 30 min, classifier accuracy ranged from 63.9% to 81.2%, and ROC-AUC ranged from 57.1% to 71.1%. Gaussian Naïve Bayes achieved the best accuracy (81.2%) and ROC-AUC (71.1%). For the balanced harmonized-128-Hz analysis, accuracy ranged from 57.9% to 66.5%, and ROC-AUC ranged from 51.3% to 69.7%. Logistic Regression also performed best in accuracy (66.5%), precision (75.3%), and ROC-AUC (64.1%), while XGBoost had the best recall (70.9%) and F1 score (63.9%), as shown in Table 11. Similarly, in Tables 12, 13, results were calculated using Harmonised-360 Hz, 30 min before balanced data; GNB achieves the highest accuracy of 80.5% after balancing, while LR achieves the highest of 67.2% among all ML classifiers. Figures 9a–c, and 10 shows the subject-level confusion matrices and overall accuracy comparison for all ML classifiers evaluated on the native and harmonized (balanced) datasets.

**Table 8:** Performance comparison of various ML classifiers (native database before balancing).

Model	Accuracy (%)	Precision (%)	Recall (%)	F1-score (%)	ROC-AUC (%)
K-Nearest Neighbors (KNN)	**81**.**0**	68.6	68.3	61.4	86.3
Gaussian Naïve Bayes	**81**.**0**	51.7	50.0	50.2	82.2
Random Forest	70.4	50.2	43.3	40.6	79.1
Support Vector Machine (SVM)	74.2	50.3	95.0	64.5	78.9
Logistic Regression	74.3	45.7	60.0	51.2	82.2
Decision Tree	74.4	57.1	66.7	52.2	76.5
Gradient Boosting	70.4	50.2	43.3	40.6	77.1
XGBoost	77.0	60.0	53.3	50.0	83.7

KNN and Gaussian Naive Bayes ML approaches yielded satisfactory accuracy (81%).

**Table 9:** Performance comparison of various ML classifiers (native database balanced).

Model	Accuracy (%)	Precision (%)	Recall (%)	F1-score (%)	ROC-AUC (%)
K-Nearest Neighbors (KNN)	**79**.**5**	78.1	89.2	81.3	85.7
Gaussian Naïve Bayes	72.7	81.9	62.7	68.2	89.4
Random Forest	71.3	76.9	70.5	69.4	83.5
Support Vector Machine (SVM)	79.0	78.6	85.7	79.8	83.0
Logistic Regression	74.0	82.5	65.5	69.8	89.4
Decision Tree	73.5	77.5	74.8	72.3	76.5
Gradient Boosting	71.3	76.9	70.5	69.4	83.1
XGBoost	79.1	81.9	80.0	78.5	84.6

KNN shows 79.5% accuracy after balancing the database.

**Table 10:** Performance comparison of various ML classifiers (harmonized-128 hz, 30 min).

Model	Accuracy (%)	Precision (%)	Recall (%)	F1-score (%)	ROC-AUC (%)
K-Nearest Neighbors (KNN)	79.2	56.6	32.1	38.4	67.4
Gaussian Naïve Bayes	81.2	62.1	29.5	38.0	71.1
Random Forest	70.2	34.0	30.5	30.4	65.0
Support Vector Machine (SVM)	70.7	37.6	45.3	38.4	62.8
Logistic Regression	66.9	33.4	46.0	37.4	59.9
Decision Tree	63.9	29.6	38.6	31.0	57.1
Gradient Boosting	70.2	34.0	30.5	30.4	63.0
XGBoost	71.2	36.7	34.7	32.9	67.3

**Table 11:** Performance comparison of various ML classifiers (harmonised-128 hz, 30 min, balanced).

Model	Accuracy (%)	Precision (%)	Recall (%)	F1-score (%)	ROC-AUC (%)
K-Nearest Neighbors (KNN)	62.9	63.0	64.9	60.9	69.7
Gaussian Naïve Bayes	62.6	71.5	43.2	51.1	64.3
Random Forest	58.6	58.2	51.2	52.2	66.5
Support Vector Machine (SVM)	57.9	60.5	42.4	46.6	51.3
Logistic Regression	66.5	75.3	49.7	56.6	64.1
Decision Tree	60.3	58.8	60.7	57.0	61.2
Gradient Boosting	58.6	58.2	51.2	52.2	62.1
XGBoost	62.9	62.1	70.9	63.9	68.2

**Table 12:** Performance comparison of various ML classifiers (harmonised-360 hz, 30 min).

Model	Accuracy (%)	Precision (%)	Recall (%)	F1-score (%)	ROC-AUC (%)
K-Nearest Neighbors (KNN)	79.2	57.9	31.8	38.4	64.3
Gaussian Naïve Bayes	80.5	54.6	26.3	33.8	71.0
Random Forest	68.0	25.8	24.4	23.5	60.2
Support Vector Machine (SVM)	69.7	38.3	48.5	40.0	62.2
Logistic Regression	67.2	33.9	48.8	38.8	60.1
Decision Tree	63.1	25.6	36.1	28.3	55.0
Gradient Boosting	68.0	25.8	24.4	23.5	59.6
XGBoost	71.0	36.0	34.5	32.5	66.7

**Table 13:** Performance comparison of various ML classifiers (harmonised-360 hz, 30 min, balanced).

Model	Accuracy (%)	Precision (%)	Recall (%)	F1-score (%)	ROC-AUC (%)
K-Nearest Neighbors (KNN)	63.1	63.4	65.3	61.5	70.1
Gaussian Naïve Bayes	62.6	71.5	43.2	51.1	64.1
Random Forest	58.7	57.8	47.8	49.8	67.2
Support Vector Machine (SVM)	58.1	62.6	43.2	47.7	51.2
Logistic Regression	67.2	75.7	51.1	57.8	64.1
Decision Tree	59.5	58.1	59.0	55.7	60.0
Gradient Boosting	58.7	57.8	47.8	49.8	61.4
XGBoost	62.4	61.6	69.9	63.1	67.9

**Figure 9:**
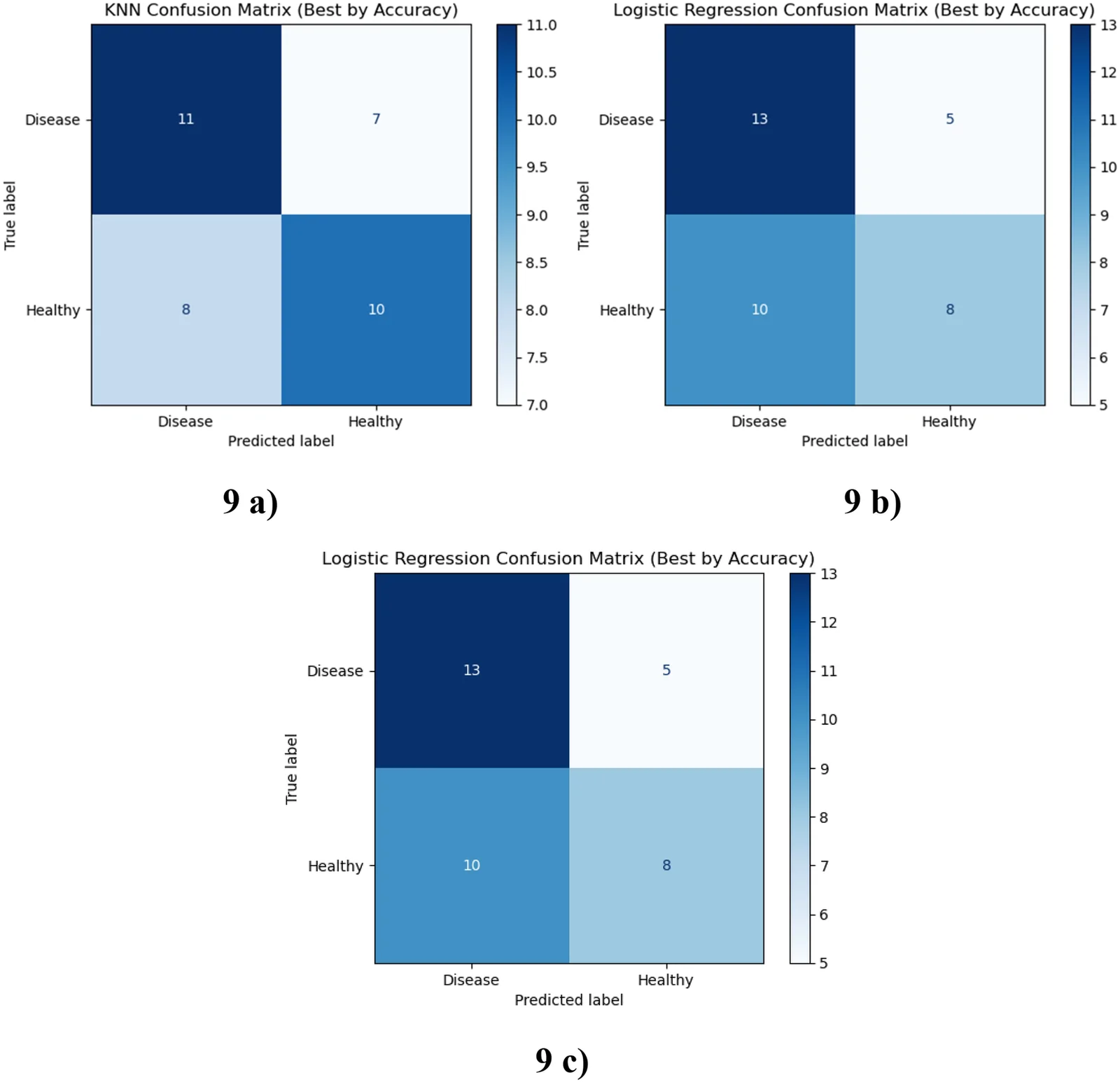
**(a–c)**. Confusion matrices for all eight classifiers under native and harmonized (128 Hz and 360 Hz).

**Figure 10:**
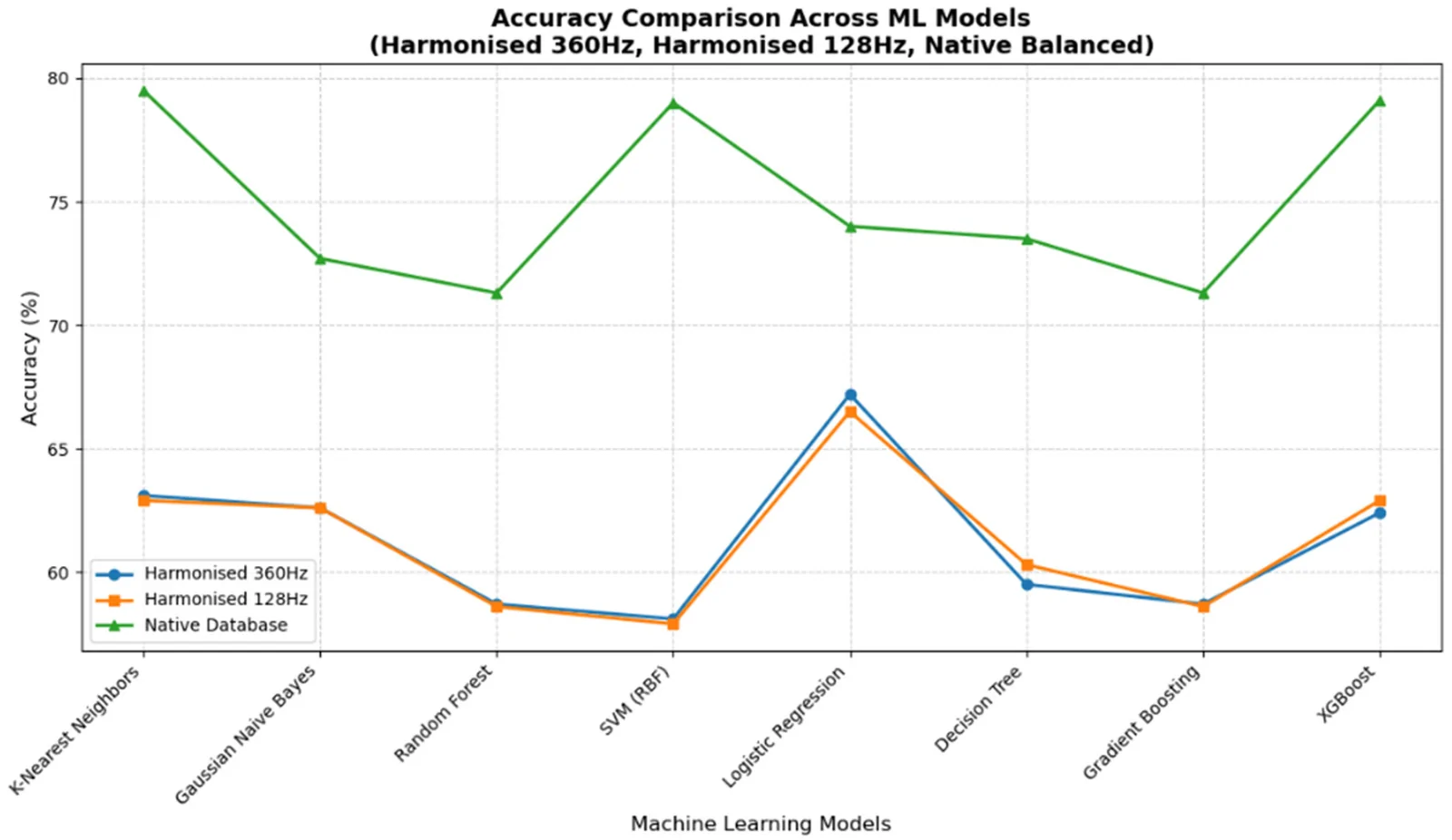
Accuracy curve of various ML classifiers (native and harmonized).

### 4.4 Diagnostic performance (native data)

The ROC analyses (Figure 11, Table 14) showed moderate to high discriminative power between all comparisons, with the highest point estimate for NSR versus PVC-dominated (AUC = 0.883, 95% CI 0.717–1.000). This comparison has a wide confidence interval, due to the small PVC subgroup, and is therefore considered preliminary.

**Figure 11:**
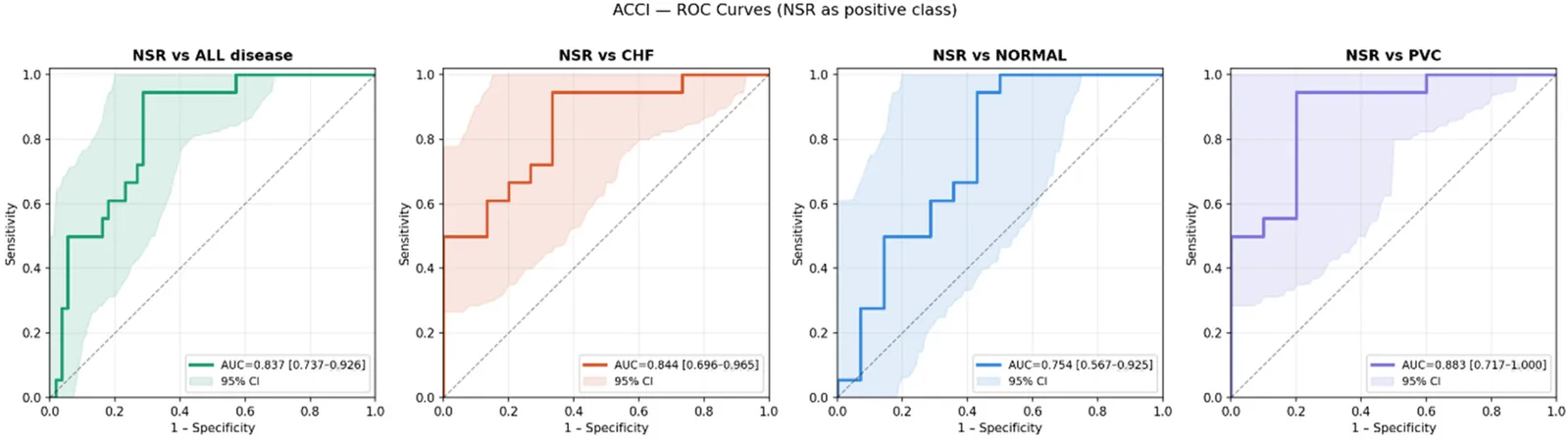
Receiver operating characteristic (ROC) curves for AVCI-based discrimination, with NSR as the positive class.

**Table 14:** Area under the curve of the receiver operating characteristic (ROC-AUC).

Comparison	n (NSR)	n (dis.)	AUC	95% CI lower	95% CI upper
NSR vs. PVC-dominant	18	10	0.883	0.717	1.000
NSR vs. CHF	18	15	0.844	0.696	0.965
NSR vs. ALL disease	18	56	0.837	0.737	0.926
NSR vs. ARR-NORMAL	18	14	0.754	0.567	0.925

### 4.5 Comparison with standard HRV metrics (native data)

Three traditional HRV measures were used to compare the discriminative performance of AVCI in differentiating between NSR and CHF. With an absolute improvement of 0.163 above the top-performing HRV metric, SDNN, AVCI scored the greatest AUC of 0.844, compared to 0.681 for SDNN, 0.567 for RMSSD, and 0.374 for mean heart rate. While mean heart rate showed an AUC below the chance level of 0.5, RMSSD demonstrated little discriminative potential. In comparison, 99.2 ms versus 92.9 ms for RMSSD and 115.3 ms versus 86.1 ms for SDNN, group-wise mean values for AVCI demonstrated better separation, with mean AVCI values of 0.663 in NSR and 0.526 in CHF. These results show that, out of all the assessed measures, AVCI offered the best observed discriminative performance. To assess whether the observed AUC differences were statistically significant, a formal statistical comparison of the associated ROC curves was carried out. RR intervals beyond the physiological range of 400–1,500 ms were not included in the HRV computation. Ectopic beats were filtered or reduced to a physiological range. Thus, Table 15 and Figure 12 describe the HRV analysis of NSR versus CHF, which do not have arrhythmia or ectopic-beat data.

**Table 15:** AUC comparison of AVCI versus standard heart rate variability metrics for NSR-versus-CHF binary discrimination.

Biomarker		AUC (NSR vs. CHF)	*Δ* AUC vs. AVCI	Interpretation
AVCI (Proposed work)		0.844	Reference	Good
HRV METRICS	SDNN	0.681	−0.163	Fair
	RMSSD	0.567	−0.277	Near chance
	Mean heart rate	0.374	−0.470	Below chance

**Figure 12:**
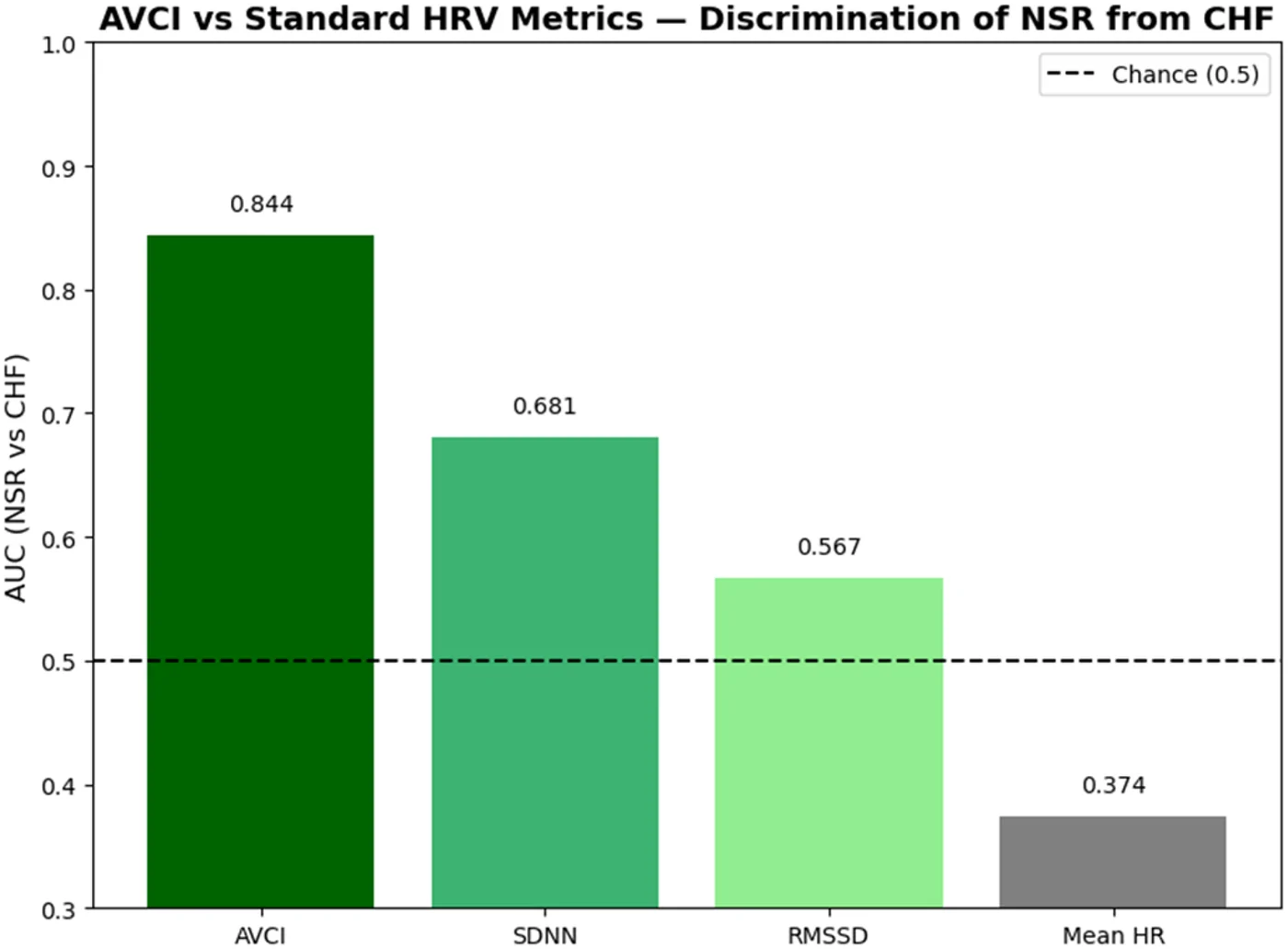
Comparison of AUC for NSR-versus-CHF discrimination across AVCI and three standard HRV metrics.

### 4.6 Classifier performance and feature importance (native data)

A Random Forest classifier was used with AVCI, SDNN, RMSSD, and average heart rate as input data. When tested with leave-one-out cross-validation, the model reached an overall accuracy of 73% and an AUC of 0.754. It correctly identified 78% of cases of normal sinus rhythm (NSR) and had a specificity of 67% in patients with congestive heart failure (CHF). SDNN (21.8%), RMSSD (16.3%), and average heart rate (16.1%) were not as significant as AVCI, which represented 45.8% of the overall feature relevance. These results show that AVCI captures key parts of heart function that traditional heart rate variability (HRV) measures do not fully capture, showing its potential as a useful, clinically relevant marker for assessing cardiovascular diseases in Figure 13 and Table 16.

**Figure 13:**
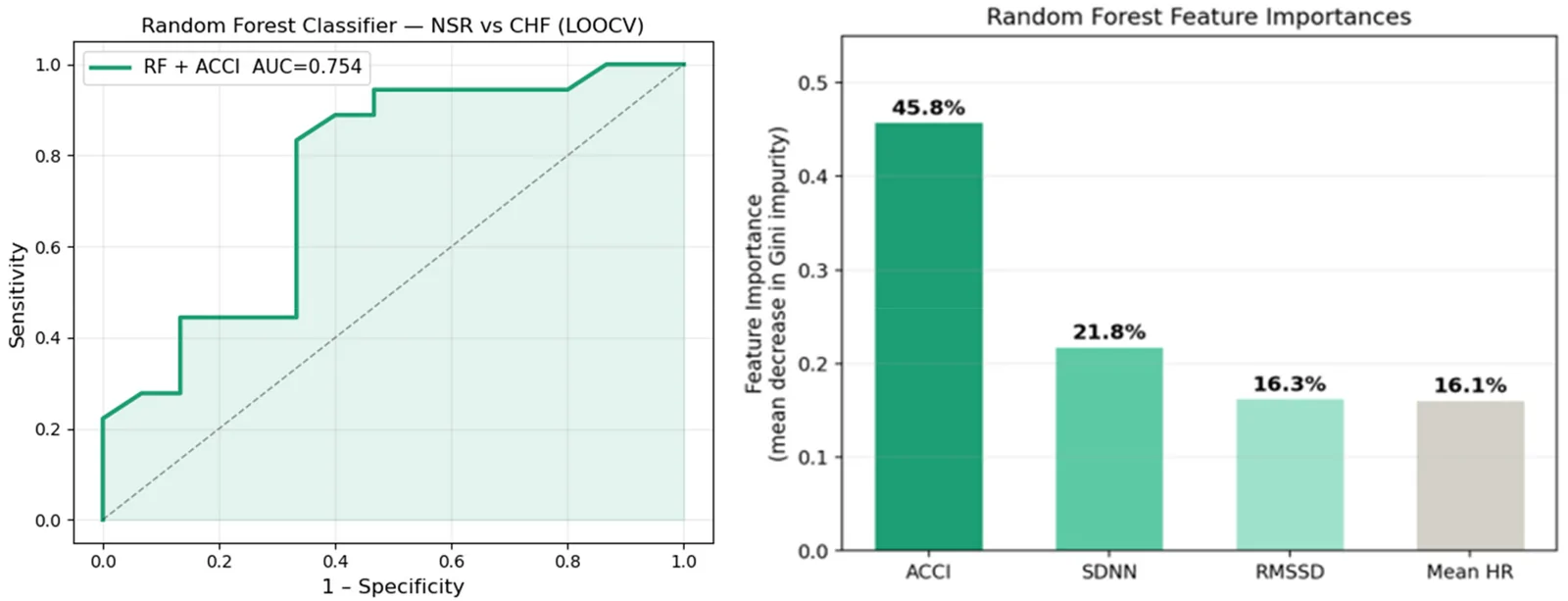
Random forest classifier for NSR-versus-CHF discrimination under leave-one-out cross-validation.

**Table 16:** Random forest leave-one-out cross-validation results for NSR-versus-CHF discrimination.

Class	Precision	Recall (Sens.)	F1-score	Support
CHF	0.71	0.67	0.69	15
NSR	0.74	0.78	0.76	18
Overall accuracy	0.73	0.73	0.73	33

### 4.7 Internal cross-validation of the AVCI threshold

To assess whether the AVCI threshold works well for people not familiar with creating it, two internal validation methods were applied to the NSR and CHF groups (*n* = 33). In stratified five-fold cross-validation, the best AVCI cut-off was found using only the training data and tested on the held-out part, resulting in an average area under the ROC curve of 0.817, accuracy of 0.757, sensitivity of 0.883, and specificity of 0.600. The best threshold stayed consistent across folds (range 0.543–0.557), closely matching the value between the NSR and CHF group averages. Another test using 200 random 70/30 train/test splits gave an average held-out AUC of 0.846 (95% range: 0.619–1.000), very close to the full-sample performance reported earlier (AUC: 0.844). The close match between the held-out cross-validated AUC (0.817–0.846) and the full-sample AUC (0.844), along with the stable decision threshold across resampling, shows that AVCI's ability to distinguish groups is not due to fitting the specific sample and that it performs well on new subjects in these groups.

### 4.8 LTDB preliminary independent validation (native data)

To test if AVCI works on completely independent data, it was calculated using the same unchanged method on the MIT-BIH Long-Term ECG Database (LTDB), which has long (14–24-hour) ambulatory recordings sampled at 128 Hz, the same rate and type as the NSR database, but from a different group with ventricular ectopy. Six recordings had enough valid data for analysis. The average AVCI in this group was 0.476 ± 0.076, clearly in the unhealthy range and much lower than the healthy NSR average (0.663 ± 0.105; Mann–Whitney U = 101, *p* = 0.0019, Cliff's delta=0.870, large effect). The LTDB group's AVCI values were similar to those of the CHF group (0.526 ± 0.095), matching the expected lower autonomic–heart cell connection in unhealthy hearts in Figure 10. This external result shows three important points for general use: AVCI can be calculated on a completely independent dataset using the same method; the values show meaningful differences rather than just noise; and healthy people are clearly separated from this independent heart group with a large effect, as expected from the physiological idea behind AVCI. Although the independent group is small, the agreement across databases supports AVCI's strength as a tool for distinguishing healthy from unhealthy heart status in Figure 14.

**Figure 14:**
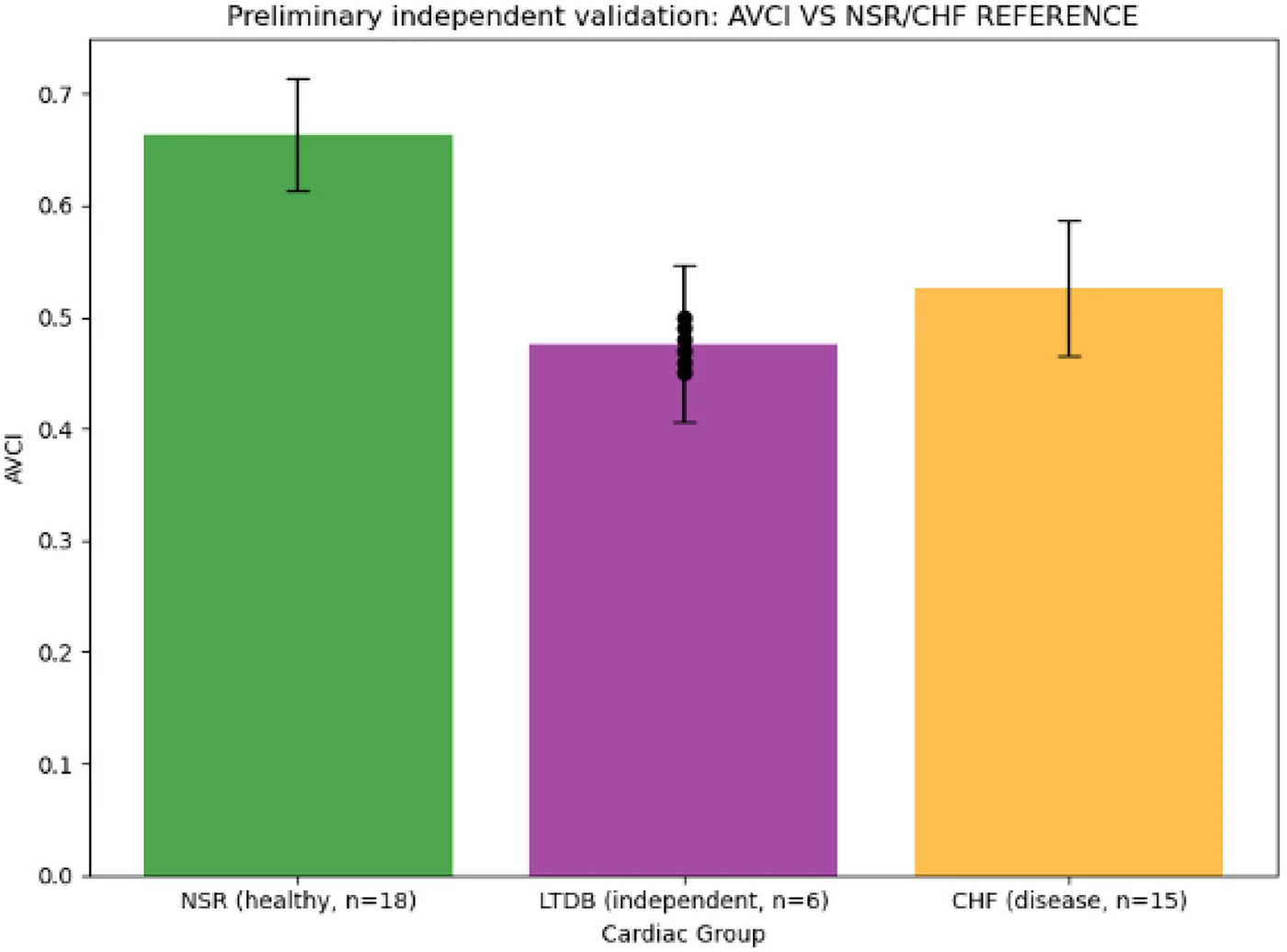
Preliminary independent validation on the independent MIT-BIH long-term ECG database (LTDB).

### 4.9 QRS-window sensitivity under harmonized conditions

Discrimination was low across all widths (NSR-vs.-CHF AUC 0.352–0.470; NSR-vs.-all-disease 0.552–0.581), establishing that this is not just a consequence of the QRS-window selection (Table 17). This is in contrast to the native condition QRS-window sensitivity (AUC 0.826- 0.859), where the duration of the recording rather than the morphology-window parameter affects the sensitivity.

**Table 17:** QRS-window sensitivity under harmonized conditions (128 hz, 30 min).

QRS (ms)	NSR mean ± SD	CHF mean ± SD	ARR mean ± SD	AUC NSR-CHF	AUC NSR-ARR	AUC NSR-all
80	0.486 ± 0.073	0.499 ± 0.093	0.478 ± 0.142	0.470	0.616	0.581
**120**	**0.488** ± **0.075**	**0.511** ± **0.088**	**0.481** ± **0.142**	**0**.**382**	**0**.**621**	**0**.**563**
150	0.483 ± 0.075	0.510 ± 0.087	0.478 ± 0.139	0.363	0.616	0.555
180	0.477 ± 0.075	0.506 ± 0.085	0.472 ± 0.138	0.352	0.616	0.552

Selected QRS-window sensitivity for our study.

## 5 Discussion

### 5.1 AVCI windows details

Table 18 summarizes AVCI computation and record inclusion and exclusion across the sampling settings. The 88 records in the collection included 7 MIT-BIH LTDB recordings utilized for external validation, 18 NSR, 15 CHF, and 48 ARR. Eight of them were not included in the AVCI computation using native sampling, whereas the remaining 80 records were. Nine records were eliminated, and 72 records were valid after harmonization to 128 Hz. Ten records were rejected, and 71 records were valid at 360 Hz. Only the native-sampling examination of the MIT-BIH LTDB recordings included six valid records and one exclusion. After resampling, NSR and CHF among the diagnostic groups displayed further exclusions, but ARR consistently had seven exclusions under all sampling conditions. Rather than the diagnostic label itself, the main cause of the exclusions was a lack of valid analysis windows for accurate AVCI estimates.

**Table 18:** The AVCI calculation of records inclusion and exclusion.

Dataset	Total records	Valid AVCI native	Excluded native	Valid AVCI 128 Hz	Excluded 128 Hz	Valid AVCI 360 Hz	Excluded 360 Hz
NSR	18	18	0	18	0	17	1
CHF	15	15	0	13	2	13	2
ARR	48	41	7	41	7	41	7
MIT-BIH LTDB (Preliminary validation)	7	6	1	–	–	–	–
Total	**88**	**80**	**8**	**72**	**9**	**71**	**10**

AVCI calculation for different records considered in our study.

### 5.2 Physiological interpretation

In healthy hearts, there is a strong coupling between RR-interval variability during respiration and QRS morphology changes in the higher AVCI in NSR. There are two physiological processes that can be associated. The respiratory sinus arrhythmia results from vagal modulation of RR-intervals at the breathing frequency, and the changes in intrathoracic pressure and venous return associated with respiration can affect the filling of the ventricle and thus modify the QRS morphology. These processes are respiratory-driven in healthy hearts and may generate coordinated oscillations in V(t) and C(t), leading to an increase in AVCI. In CHF, the lower mean AVCI (0.663 in NSR vs. 0.526 in CHF; Cliff's delta = 0.689) could result from reduced vagal activity, increased sympathetic influence, and ventricular remodeling. Frequent PVCs result in high intraventricular activation irregularity and changes in QRS morphology that may not be well synchronized with V(t), and this effect size is the highest (Cliff's delta = 0.767). The autonomic–ventricular coupling, however, is still a physiological hypothesis, the validity of which should be tested with simultaneous measurements of respiration, autonomic activity, and ventricular mechanics.

### 5.3 Machine learning interpretation

Deployment Practicality: The machine learning evaluation further demonstrates the feasibility of using the suggested AVCI method in actual healthcare settings. Using very simple learning algorithms, KNN, Gaussian Naïve Bayes, and Logistic Regression achieved likely classification performance, making them appropriate for use in contexts with limited computing power. The suggested framework is ideal for point-of-care decision support and real-time cardiac monitoring since AVCI is obtained from a single-lead ECG without requiring complicated waveform characterization. Integration into wearable ECG devices, bedside patient monitoring systems, remote telemedicine platforms, and other healthcare settings with minimal resources is made possible by the low processing needs. The suggested AVCI architecture provides a workable and scalable approach for ongoing cardiac risk assessment and early identification of cardiovascular problems by fusing physiological interpretability with effective machine learning categorization.

### 5.4 Advantages over standard HRV

The AUC comparison shows that AVCI performs much better than SDNN, RMSSD, and average heart rate at distinguishing NSR from CHF. This is especially clear for RMSSD, which, although commonly used to measure parasympathetic function, performed almost no better than chance here because the average values were nearly the same in both groups. Standard HRV measures only the timing changes of RR intervals; AVCI, however, measures the link between autonomic changes and ventricular shape, capturing an additional aspect of heart function. The Random Forest analysis supports this, showing that AVCI is more important than the combined effect of all three HRV measures. These findings position AVCI as a complement to conventional HRV rather than a replacement.

### 5.5 Comparison with prior coherence-based methods

Existing coherence-based ECG methods have examined relationships between ECG waveform components or between ECG and external physiological signals (12, 14). In contrast, AVCI is derived from a single ECG lead by measuring spectral coherence between RR-interval changes and QRS-area changes. It does not require multiple leads, respiratory signals, or manual identification of specific ECG segments, and can be computed from recordings sampled at moderately low rates, such as 128 Hz. To the best of our knowledge, this specific RR–QRS-area coherence relationship has not previously been proposed as a single-lead ECG method.

### 5.6 Limitations

The small size of the population where AVCI was evaluated was due to limited availability of long-duration Holter recordings. This analysis was performed on retrospective review databases of ECGs, which could have been affected by varying recording conditions, patient characteristics, and quality of the ECG. For additional validation, standardized acquisition protocols and larger, prospectively defined populations are needed. Artifacts, noise, and preprocessing procedures can influence feature extraction and coherence estimation, since this is done by AVCI based on RR intervals and QRS areas. The robustness and the reproducibility of AVCI need to be evaluated further and under different preprocessing and signal-quality conditions. NSR and CHF recordings made up most of the arrhythmia database recordings, which were considerably shorter (about 30 min) than recordings made with the NSR and CHF, and thereby had fewer windows of analysis and possibly less statistical power. A difference in the recording duration could have been a factor in the lack of significant difference between NSR and ARR-NORMAL following correction. There are also several limitations: (1) the development groups were drawn from different databases with differing acquisition characteristics; (2) the within-database standardized analysis did not demonstrate discrimination beyond chance; (3) sufficient recording duration is required for the use of AVCI, which may restrict its usefulness in short clinical ECG recordings; (4) measurements and analysis of respiration, autonomic activity and ventricular mechanical function did not directly validate the proposed physiological mechanism; (5) the AF and PAC subgroups consisted of only two recordings and were reported descriptively; and (6) Since direct measurements of respiration, autonomic activity, and ventricular mechanical function were not included in the recordings, the suggested physiological explanation is speculative. As a result, AVCI is still regarded as a technique that needs more clinical and disease-specific validation.

### 5.7 Clinical and practical implications

Some of the most important practical advantages of the AVCI are its ease of computation, ease of interpretation, and the use of a single ECG lead, making it well suited to low-cost and automated screening environments**.** AVCI offers several advantages for clinical use when collected from long-term databases with consistent harmonization. It is simple to apply across a variety of healthcare settings because it requires only a single ECG lead, no additional patient data, and manual heartbeat marking. The technique is quick on a standard computer, analyzing a 24-hour ECG recording in less than two minutes. These characteristics make AVCI compatible with mobile cardiac monitors, wearable ECG devices, and older Holter recordings. AVCI offers a distinct, single-value marker from the ECG signal and illustrates the relationship between the nervous system and heart cells, in contrast to many deep learning ECG models. It is helpful in clinical practice, regulatory evaluation, and routine heart risk assessments because of its straightforward methodology.

### 5.8 Future validation

The crucial next step would be to acquire uniform, long-duration single-lead ECG recordings from a single population of healthy and diseased individuals, preferably with simultaneous respiration and independent autonomic and ventricular-mechanical measurements. A design of this sort would isolate differences in the computational association measured by AVCI from differences in acquisition and population and would properly test the proposed physiological interpretation.

## 6 Conclusion

The Autonomic–Ventricular Coherence Index (AVCI) is a simple and interpretable AV coupling index that can be derived from a single ECG lead. It exhibited a good discrimination value in native conditions and a moderate discrimination value in harmonized acquisition, similar to the traditional HRV measures. The machine learning models also strengthened AVCI's potential, with promising results under native and moderate harmonization conditions. There was a high degree of accuracy in R-peak detection, and the results were consistent over various QRS windows. The study reveals that overall, acquisition parameters such as the sampling rate and the length of the recording are key to the diagnostic performance of AVCI. AVCI integrates coherence-based biomarkers with machine learning, offering a roadmap for more powerful and explainable cardiovascular diagnostics. Standardized acquisition protocols will be crucial for facilitating the clinical use of AVCI and will maximize its clinical potential.

## Data Availability

The original contributions presented in the study are included in the article/Supplementary Material, further inquiries can be directed to the corresponding author.

## References

[1] World Health Organization. World Health Organization. Cardiovascular diseases. Available online at: https://www.who.int/health-topics/cardiovascular-diseases#tab=tab_1 (Accessed July 8, 2026).

[2] India: health of the nation’s states: the India state-level disease burden initiative: disease burden trends in the states of India, 1990 to 2016. Indian Council of Medical Research. (2017). p. 214.

[3] Potter JM Hickman PE Cullen L. Abnormal laboratory results: troponins in myocardial infarction and injury. Aust Prescr. (2022) 45(2):53–7. 10.18773/austprescr.2022.006 35592367 PMC9081942

[4] Califf RM. Biomarker definitions and their applications. Exp Biol Med. (2018) 243(3):213–21. 10.1177/1535370217750088 PMC5813875 29405771

[5] Burlacu A Brinza C Geman O Karppa M Hemanth DJ. Heart rate variability as a dual-use digital biomarker: integrating clinical, AI, and operational perspectives on human performance and resilience. BMC Cardiovasc Disord. (2026) 26(1):87. 10.1186/s12872-026-05543-z 41580586 PMC12849089

[6] Yan SP Song X Wei L Gong YS Hu HY Li YQ. Performance of heart rate adjusted heart rate variability for risk stratification of sudden cardiac death. BMC Cardiovasc Disord. (2023) 23(1):144. 10.1186/s12872-023-03184-0 36949420 PMC10032001

[7] Huikuri HV. Heart rate variability in coronary artery disease. J Intern Med. (1995) 237(4):349–57. 10.1111/j.1365-2796.1995.tb01186.x 7714457

[8] Laborde S Mosley E Thayer JF. Heart rate variability and cardiac vagal tone in psychophysiological research – recommendations for experiment planning, data analysis, and data reporting. Front Psychol. (2017) 08. 10.3389/fpsyg.2017.00213 PMC5316555 28265249

[9] Wang J Guo X. A deep learning strategy for automatic congestive heart failure detection using novel bottleneck attention module. Appl Intell. (2024) 54(17–18):8120–31. 10.1007/s10489-024-05364-7

[10] Sharma K Rao BM Marwaha P Kumar A. Accurate detection of congestive heart failure using electrocardiomatrix technique. Multimed Tools Appl. (2022) 81(21):30007–23. 10.1007/s11042-022-12773-8

[11] Sinha N Das A. Identification and localization of myocardial infarction based on analysis of ECG signal in cross spectral domain using boosted SVM classifier. IEEE Trans Instrum Meas. (2021) 70:1–9. 10.1109/TIM.2021.311766333776080

[12] Liao F Li Y Lyu S Chen P Yu-Ju Hung I Pappu S. Using wavelet phase coherence of heart rate variability and blood flow oscillations to compare mechanisms of action between tai chi mind-body exercise and brisk walking aerobic exercise. Biomed Signal Process Control. (2024) 95:106385. 10.1016/j.bspc.2024.106385

[13] Penzel T Kantelhardt JW Bartsch RP Riedl M Kraemer JF Wessel N. Modulations of heart rate, ECG, and cardio-respiratory coupling observed in polysomnography. Front Physiol. (2016) 7. 10.3389/fphys.2016.00460 27826247 PMC5078504

[14] Piccirillo G Moscucci F Persi A Di Barba D Pappadà MA Rossi P. Intra-QT spectral coherence as a possible noninvasive marker of sustained ventricular tachycardia. Biomed Res Int. (2014) 2014:1–9. 10.1155/2014/583035 PMC4123476 25133170

[15] Sathi TA Jany R Ela RZ Azad AKM Alyami SA Hossain MA. An interpretable electrocardiogram-based model for predicting arrhythmia and ischemia in cardiovascular disease. Results Eng. (2024) 24:103381. 10.1016/j.rineng.2024.103381

[16] Aziz S Ahmed S Alouini MS. ECG-based machine-learning algorithms for heartbeat classification. Sci Rep. (2021) 11(1):18738. 10.1038/s41598-021-97118-5 34548508 PMC8455569

[17] Karri M Annavarapu CSR. A real-time embedded system to detect QRS-complex and arrhythmia classification using LSTM through hybridized features. Expert Syst Appl. (2023) 214:119221. 10.1016/j.eswa.2022.119221

[18] Huang ML Wu YS. Classification of atrial fibrillation and normal sinus rhythm based on convolutional neural network. Biomed Eng Lett. (2020) 10(2):183–93. 10.1007/s13534-020-00146-9 32431951 PMC7235126

[19] Han Z Li B Liu J. Predictive value of machine learning for mortality risk in aortic dissection: a systematic review and meta-analysis. Int J Med Inform. (2026) 209:106271. 10.1016/j.ijmedinf.2026.106271 41539126

[20] Jayaraman Rajendiran DK Ganesh Babu C Priyadharsini K Karthi SP. Certain investigation on hybrid neural network method for classification of ECG signal with the suitable a FIR filter. Sci Rep. (2024) 14(1):15087. 10.1038/s41598-024-65849-w 38956261 PMC11219891

[21] Abbaszadeh A Bazargani M. Heart disease prediction using ECG-based lightweight system in IoT based on meta-heuristic approach. Heliyon. (2024) 10(23):e40537. 10.1016/j.heliyon.2024.e40537 39669140 PMC11636128

[22] Mantri N Lu M Zaroff JG Risch N Hoffmann T Oni-Orisan A. QT interval dynamics and cardiovascular outcomes: a cohort study in an integrated health care delivery system. J Am Heart Assoc. (2021) 10(19). 10.1161/JAHA.120.018513 34581201 PMC8649135

[23] Tikkanen JT Kentta T Porthan K Anttonen O Eranti A Aro AL. Risk of sudden cardiac death associated with QRS, QTc, and JTc intervals in the general population. Heart Rhythm. (2022) 19(8):1297–303. 10.1016/j.hrthm.2022.04.016 35472593

[24] Aufiero S Bleijendaal H Robyns T Vandenberk B Krijger C Bezzina C. A deep learning approach identifies new ECG features in congenital long QT syndrome. BMC Med. (2022) 20(1):162. 10.1186/s12916-022-02350-z 35501785 PMC9063181

[25] Plagwitz L Doldi F Magerfleisch J Zotov M Bickmann L Heider D. QTcNet: a deep learning model for direct heart rate corrected QT interval estimation. Europace. (2025) 27(12). 10.1093/europace/euaf274 41140122 PMC12696413

[26] Singh AK Krishnan S. Trends in EEG signal feature extraction applications. Front Artif Intell. (2023) 5. 10.3389/frai.2022.1072801 36760718 PMC9905640

[27] Mincholé A Zacur E Ariga R Grau V Rodriguez B. MRI-based computational torso/biventricular multiscale models to investigate the impact of anatomical variability on the ECG QRS complex. Front Physiol. (2019) 10. 10.3389/fphys.2019.01103 PMC6718559 31507458

[28] Madona P Basti RI Zain MM. PQRST wave detection on ECG signals. Gac Sanit. (2021) 35:S364–9. 10.1016/j.gaceta.2021.10.052 34929852

[29] Moody GB. MIT-BIH Normal Sinus Rhythm Database. (1999). Available online at: https://www.physionet.org/content/nsrdb/1.0.0/ (Accessed July 9, 2026).

[30] Beth Israel Deaconess Medical Center/PhysioNet. BIDMC Congestive Heart Failure Database. (2000). Available online at: https://www.physionet.org/content/chfdb/1.0.0/ (Accessed July 9, 2026).

[31] Moody GB. MIT-BIH Arrhythmia Database. (2005). Available online at: https://www.physionet.org/content/mitdb/1.0.0/ (Accessed July 9, 2026).

[32] Hamilton PS Tompkins WJ. Quantitative investigation of QRS detection rules using the MIT/BIH arrhythmia database. IEEE Trans Biomed Eng. (1986) BME-33(12):1157–65. 10.1109/TBME.1986.325695 3817849

[33] Li Q Liu Y Zhao N Yuan Y He R. A novel ECG QRS complex detection algorithm based on dynamic Bayesian network. Artif Intell Med. (2026) 174:103370. 10.1016/j.artmed.2026.103370 41653675

[34] Moody GB. MIT-BIH Long-Term ECG Database. (1999). Available online at: https://physionet.org/content/ltdb/1.0.0/ (Accessed July 9, 2026).

[35] Chicco D Karaiskou AI De Vos M. Ten quick tips for electrocardiogram (ECG) signal processing. PeerJ Comput Sci. (2024) 10:e2295. 10.7717/peerj-cs.2295 39314696 PMC11419615

[36] IEEE. IEEE Technology Navigator. Signal Sampling. Available online at: https://technav.ieee.org/topic/signal-sampling/ (Accessed July 8, 2026).

[37] Merino-Monge M Castro-García JA Lebrato-Vázquez C Gómez-González IM Molina-Cantero AJ. Heartbeat detector from ECG and PPG signals based on wavelet transform and upper envelopes. Phys Eng Sci Med. (2023) 46(2):597–608. 10.1007/s13246-023-01235-6 36877361 PMC10209290

[38] Pan J Tompkins WJ. A real-time QRS detection algorithm. IEEE Trans Biomed Eng. (1985) BME-32(3):230–6. 10.1109/TBME.1985.325532 3997178

[39] Ahmed AF. A novel improved Pan-Tompkins method for QRS complex detection of the ECG signal. Signal Image Video Process. (2025) 19(12):960. 10.1007/s11760-025-04587-6

[40] Alhalabi A Alzahrani S Tamal M. Robust and parameter free QRS complex detection in ECG for heart disease diagnosis and monitoring. Signal Image Video Process. (2025) 19(12):986. 10.1007/s11760-025-04588-5

[41] Habibi Z Karimizadeh K Nosratpour A Alipourfard I. Enhanced QRS detection and ECG compression using adaptive thresholding: a real-time approach for improved monitoring and diagnosis. Comput Electr Eng. (2024) 119:109528. 10.1016/j.compeleceng.2024.109528

[42] Jebastine J. Fetal ECG extraction and QRS detection using advanced adaptive filtering-based signal decomposition and peak threshold technique from abdominal ECG signals. Circuits Syst Signal Process. (2023) 42(10):6058–88. 10.1007/s00034-023-02386-3

[43] Ledezma CA Altuve M. Optimal data fusion for the improvement of QRS complex detection in multi-channel ECG recordings. Med Biol Eng Comput. (2019) 57(8):1673–81. 10.1007/s11517-019-01990-3 31098721

